# Ecological impacts of photosynthetic light harvesting in changing aquatic environments: A systematic literature map

**DOI:** 10.1002/ece3.8753

**Published:** 2022-03-22

**Authors:** Nils Hendrik Hintz, Brian Schulze, Alexander Wacker, Maren Striebel

**Affiliations:** ^1^ Institute for Chemistry and Biology of the Marine Environment (ICBM) Carl von Ossietzky University of Oldenburg Wilhelmshaven Germany; ^2^ Zoological Institute and Museum University of Greifswald Greifswald Germany

**Keywords:** climate change, ecological competition, phytoplankton, primary production, systematic map, underwater light

## Abstract

Underwater light is spatially as well as temporally variable and directly affects phytoplankton growth and competition. Here we systematically (following the guidelines of PRISMA‐EcoEvo) searched and screened the published literature resulting in 640 individual articles. We mapped the conducted research for the objectives of (1) phytoplankton fundamental responses to light, (2) effects of light on the competition between phytoplankton species, and (3) effects of climate‐change‐induced changes in the light availability in aquatic ecosystems. Among the fundamental responses of phytoplankton to light, the effects of light intensity (quantity, as measure of total photon or energy flux) were investigated in most identified studies. The effects of the light spectrum (quality) that via species‐specific light absorbance result in direct consequences on species competition emerged more recently. Complexity in competition arises due to variability and fluctuations in light which effects are sparsely investigated on community level. Predictions regarding future climate change scenarios included changes in in stratification and mixing, lake and coastal ocean darkening, UV radiation, ice melting as well as light pollution which affect the underwater light‐climate. Generalization of consequences is difficult due to a high variability, interactions of consequences as well as a lack in sustained timeseries and holistic approaches. Nevertheless, our systematic literature map, and the identified articles within, provide a comprehensive overview and shall guide prospective research.

## Graphical abstract

Underwater light is spatially and temporally variable in its intensity (quantity) and spectrum (quality) with species‐specific consequences for phytoplankton. Climate change further affects the light availability in aquatic ecosystems and consequently phytoplankton ecology. Here, we systematically mapped previously published literature to provide an extensive overview of trends and gaps in research, covering the effects of light on phytoplankton.

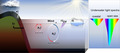

## 1 INTRODUCTION

Light is of major biological relevance as a fundamental resource for photosynthetic organisms. By absorbing the light's photosynthetic active radiation (PAR, in the wavelength range of 400–700 nm) and exploiting its energy through photosynthesis, phytoplankton contributes to approximately half of the earth's primary production, provides oxygen and energy as well as nutrients for higher trophic levels (Dokulil & Kaiblinger, 2009; Falkowski, 2012; Field et al., 1998; Martin et al., 2018). Within aquatic ecosystems, the availability of light was found to affect primary production, structure phytoplankton communities, and therefore indirectly affect higher trophic levels (see e.g., Kirk, 2010). However, light cannot only be seen as a resource of energy, but also needs to be considered as a cell damaging, photosynthesis inhibiting, and metabolism regulating factor (Ragni, 2004; Straka & Rittmann, 2018). Here, in contrast to previous reviews, we aimed to systematically map the previously conducted research on the effects of light in phytoplankton ecology. This shall provide researchers of (1) a thematic overview, (2) estimations of the extent to which an issue has been investigated, (3) reveal open gaps in research, and (4) provide a solid list of references covering the topic. We investigated the ecological impacts of light, its variability in aquatic ecosystems and displayed future scenarios. To provide a systematic overview, we split this topic into three main objectives (O) as follows:

1. O1 The fundamental ecological responses of phytoplankton to:
  - the underwater light‐climate.
  - changes and variability in light‐climate.
  - fluctuations in the light‐climate.
2. O2 Competition for light and vertical arrangement of phytoplankton in (non‐static) light gradients.
3. O3 Ecological effects of light‐climate changes on phytoplankton under future predictions of:
  - ocean and lake stratification as well as changing mixing conditions.
  - lake and coastal ocean darkening.
  - UV radiation impact.
  - melting sea ice.
  - light pollution.

These were analyzed according to the guideline of the Preferred Reporting Item for Systematic Reviews and Meta‐analysis in Ecology and Evolutionary biology (PRISMA‐EcoEvo) (O'Dea et al., 2021) and the concept as well as these objectives were pre‐registered after an initial literature search at OSF‐Registries (https://osf.io/ky3ut). We conducted comprehensive electronic searches for published resources in Web of Science on 15th of June 2021 covering all published data for each of the three main objectives. Suitable articles were identified by “topic,” that is, keywords in titles, abstracts, and author keywords of those records. The resulting records were imported to EndNote version X8 (Clarivate). Unsuitable records were excluded by title screening and subsequent abstract screening. Additional useful and criteria fitting articles which were known to the authors, listed in articles reference list, or identified within the respective other objectives, were added manually. (See Appendix S1 for detailed information on search terms and conditions, Appendix S2 for PRISMA‐like flow chart of report screening, and Appendix S3 for the PRISMA‐EcoEvo checklist.)

The search resulted in a total of 3357 records (Objective 1 (O1): 2138; Objective 2 (O2): 241; and Objective 3 (O3): 978). With inclusion of additional articles, a total of 675 records (O1: 361; O2: 59; O3: 255), that is, 640 individual articles due to duplication across the objectives, were retrieved and considered for mapping (Table 1). The median year of publication was used to roughly estimate trends in research.

**TABLE 1:** Overview of literature search and mapping results

Objective	Identified articles (median year of publication)	Trends and/or knowledge gaps
(O1) The phytoplankton's fundamental ecological responses to: the underwater light‐climate changes and variability in light‐climate fluctuations in the light‐climate	**Entire objective 362 (2008)**	Overall trend toward more realistic environmental considerations by acknowledging the spectrum and variability of light. More research needed which considers timescales and amplitudes as well additional changes in spectrum of light fluctuation.
	Intensity w/o spectrum 229 (2004) Spectrum 133 (2013) for biotechnological purpose 35 (2017)	
	Sensing 11 (2014)	
	Acclimation 154 (2009) to intensity w/o spectrum 101 (2008) to spectrum 53 (2012)	
	Regulation 24 (2007)	
	Adaptation 26 (2011)	
	Protection 49 (2012)	
	Light fluctuations 98 (2005) for biotechnological purpose 21 (2013)	
	Vertical mixing 38 (2001)	
(O2) Competition for light and vertical arrangement of phytoplankton in (non‐static) light gradients	**Entire objective 59 (2009)**	More research needed on community level. Self‐shading and feedbacks in acclimation need to be further investigated in terms of vertical arrangement.
	Vertical arrangement 15 (2009)	
(O3) Ecological effects of light‐climate changes on phytoplankton under future predictions of: ocean and lake stratification as well as changing mixing conditions lake and coastal ocean darkening UV radiation impact melting sea ice light pollution	**Entire objective 255 (2013)**	Difficult predictions due to interaction of climate change induced effects and lack of sustained time series. Generalization of consequences for phytoplankton is difficult due to high spatial and temporal variability. Lack of data for effects of different UV subtypes. Insufficiently investigation of light pollution in aquatic environments.
	Stratification and mixing 89 (2012)	
	Lake and coastal ocean darkening 85 (2014)	
	UV radiation 78 (2014) w/o differentiation of UV subtypes 34 (2014) only considering UV‐B 16 (2012)	
	Melting sea ice 32 (2014)	
	Light pollution 4 (2016)	

Note

Results are sorted by each of the three objectives as well as topics within. The number of identified articles is stated with its respective topic as well as the median year of publication in brackets. Bold numbers indicate results for the whole objective search. Additionally, identified trends and open knowledge gaps are shortly summarized.

## 2 THE PHYTOPLANKTON'S FUNDAMENTAL ECOLOGICAL RESPONSE TO THE UNDERWATER LIGHT‐CLIMATE

In aquatic environments, the light's intensity exponentially attenuates with water depth and its spectrum changes due to the selective absorption of photons by water molecules (Kirk, 2010). In a clear water column, the long (red range) wavelengths of the PAR are absorbed the most and the remaining light spectrum changes in a gradient with increasing depth toward green–blue at medium depth and blue at the bottom of the euphotic zone. Additional light‐spectrum alterations emerge by colored or chromophoric dissolved organic matter (cDOM, gilvin), particles such as detritus and sediments (tripton) as well as living organisms (primarily phytoplankton itself) (Kirk, 2010). Those are not only attenuating the light intensity but further shifting the light‐spectrum, as, for example, cDOM in general absorbs light stronger at low wavelengths below 500 nm resulting in “brownificated” water (Coble, 2007; Markager et al., 2004). Likewise, intense algae growth can shift the underwater light spectrum “greenish” due to its relative low absorbance of green light (Leech et al., 2018). In ecological research, the light is therefore often either considered in terms of its integrated intensity (quantity) or its spectral composition (quality). However, as both affect the phytoplankton, future research has to consider both aspects simultaneously.

### 2.1 Underwater light‐climate

**FIGURE 1:**
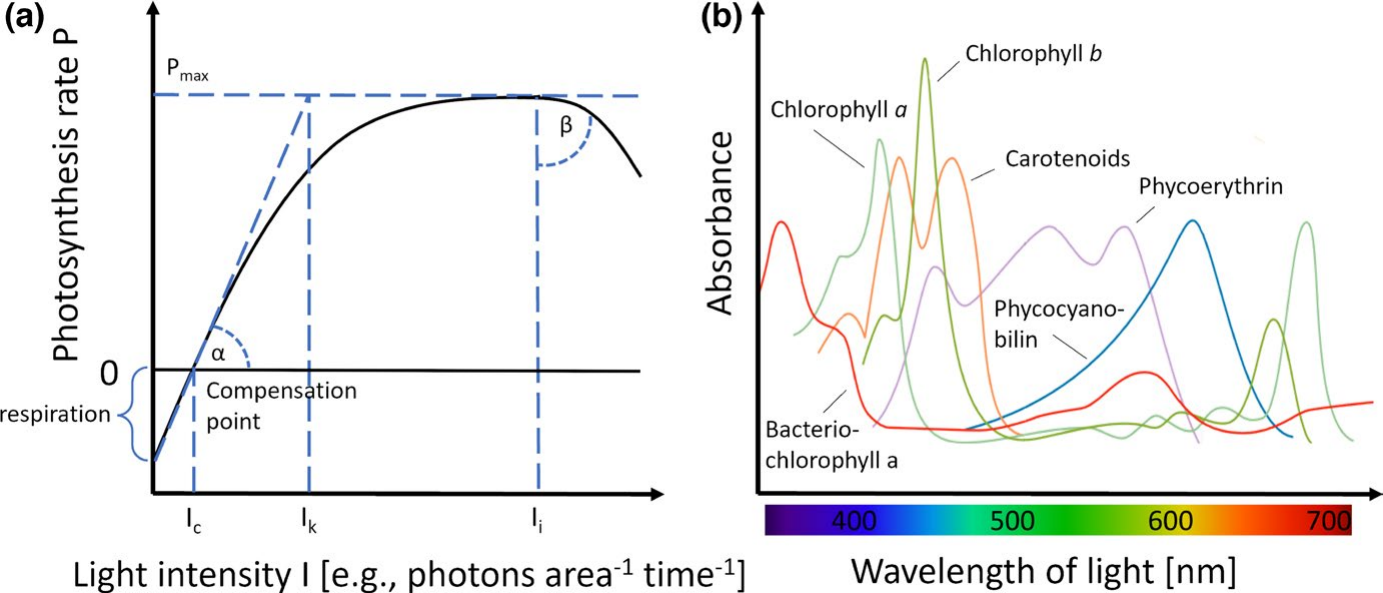
(a) Generalized photosynthesis–irradiance (*P*–*I*) curve showing the photosynthetic response (*P*) to light intensity (*I*). Thereby a positive net photosynthesis (gross photosynthesis–respiration) can lead to a positive growth rate of a phototroph. At light intensities below the compensation point, it is not sufficient to equal respiration and net photosynthesis is negative. At light intensity above the compensation point, the initial slope (*α*) of photosynthesis is limited to a maximum (*P*_max_) due to saturation. Per definition, saturation sets in at an intensity (*I*_k_) at which a linear growth of the slope *α* would reach *P*_max_. At high light intensities (*I*_p_), photoinhibition sets in as the photosynthetic apparatus becomes damaged to a certain degree (*β*). Modified after Lalli and Parson (1997) and Dokulil and Kaiblinger (2009). (b) Schematic overview of different absorption spectra of selected groups of pigments. Those can extend the light absorption to wavelengths which are less covered by chlorophyll *a*. Absorbance values are not for scale. Modified after Voet and Voet (2010)

The direct consequences of light, only in its integrated intensity (quantity) as a single resource that determines growth, were considered in 229/362 articles (within O1, which covered light exclusively in terms of intensity). It is acknowledged that the growth rates of phytoplankton increase with light intensity, driven by photosynthesis as long as other resources (such as nutrients) are not limited. Photosynthesis is nonlinear related to light supply, can be visualized as photosynthesis‐irradiance (*P*–*I*) curve, and described by three distinct regions (Dokulil & Kaiblinger, 2009; Kirk, 2010; Lalli & Parson, 1997) (Figure 1a): (1) When light is limiting, the rate of photosynthesis increases with higher light intensity. Above the compensation point, where photosynthesis equals the respiration of the cells, photosynthesis increases with intensity until it becomes limited by saturation of the photosynthetic apparatus. (2) The light saturated region indicates the light intensity in a range from the saturation onset point until the onset of photoinhibition. (3) At very high‐light intensities, the photosynthetic apparatus can become damaged by, for example, shrinking of chloroplasts, which results in reduced photosynthesis rates (Lalli & Parson, 1997).

Of such a *P*–*I* curve, one can deviate species‐specific traits of phytoplankton: The required light intensity (*I*_c_) at the compensation point and at which the initial slope (*α*) of the curve describes the efficiency of maximum quantum yield of photosynthesis (Dokulil & Kaiblinger, 2009). The maximum photosynthesis rate (*P*_max_) and the onset point of saturation (*I*_k_) at which photon absorption exceeds the electron transport of the photosynthetic apparatus (Dokulil & Kaiblinger, 2009). And the intensity at which photoinhibition starts (*I*_i_) as well as the degree of inhibition (*β*) which describes how well a phototroph can cope with damaging effects of light (Lalli & Parson, 1997).

All these traits were found to be species specific, have a high interspecific variation and correlate with other phytoplankton traits, for example, with a lower α at a larger cell size (Edwards et al., 2015). Falkowski and Owens (1978) found, for example, that the compensation light intensity can vary over four orders of magnitude between species. This implies that a phytoplankton species that still has positive net photosynthesis and growth rates at a low‐light intensity has a competitive advantage over a species that is not capable of positive rates at that light intensity (Edwards et al., 2015; Huisman & Weissing, 1994; Weissing & Huisman, 1994). Estimated species‐specific *P*–*I* curves and growth responses to the light intensity can therefore be used as predictor for competitive outcomes (Huisman et al., 1999; Huisman & Weissing, 1994; Weissing & Huisman, 1994). Additionally, there are tradeoffs among these traits, for example, species that grow well at low irradiance grow poorly at high irradiance and vice versa (Falkowski, 1980; Litchman, 2007; Richardson et al., 1983; Ryther, 1956). Along the vertical light gradient in aquatic ecosystems, such tradeoffs can therefore lead to niche separation and result in different community compositions at different depths (Schwaderer et al., 2011).

Additionally, we identified 133/362 articles which considered the effects of the light's spectrum on photosynthesis and growth, as different wavelengths are exploited species specifically. Phytoplankton harvests the light via absorption by their pigments built into the light harvesting complex (LHC), often referred to as antennae complex (Kirk, 2010). Most eukaryotic phytoplankton species rely on LHCs characterized by the combination of a central chlorophyll *a* molecule and accessory pigments belonging to the groups of chlorophylls and carotenoids (Jeffrey & Wright, 2006). In contrast, cyanobacteria, red algae, and glaucophytes feature phycobilisomes as LHCs equipped with phycobiliproteins as light absorbing pigments (Glazer, 1985). These pigments absorb the available wavelengths of the PAR with a different efficiency (Figure 1b). The chlorophylls are characterized by two absorption peaks in the blue (~440 nm) and red wavelength range (~650 nm) but only a low absorption in the green (~500–600 nm) part of the spectrum (Jeffrey & Wright, 2006). The carotenoids are a very diverse pigment group which in general absorb in the blue–green region (~300–500 nm) (Kirk, 2010). They extend the absorption range of the chlorophylls and are further involved in photoprotective mechanisms by non‐photochemical quenching of excessive energy (Brunet et al., 2011) (see also below). The phycobiliproteins phycoerythrin, phycocyanin, and allophycocyanin efficiently absorb green (~565 nm), yellow (~620 nm), and red (~650 nm) wavelengths, respectively (Grossman et al., 1993). Besides the ubiquitous chlorophyll *a*, the occurrence and composition of those accessory pigments varies remarkedly among different species (Kirk, 2010). Certain combinations of pigments result in light absorptions that allows species to absorb portions of PAR with varying efficiency, which, in turn, affects species performance and competition (Glover et al., 1987; Luimstra et al., 2019).

However, the sum of the absorption of individual pigments (i.e., individually extracted pigments) does not accurately/directly determine the light harvesting of the phytoplankton, because the pigment absorption is slightly different due to (1) bindings in pigment–protein complexes and (2) self‐shading of the pigments in the chloroplast, known as package effect (Kirk, 2010). Furthermore, differences in the optical properties of the organisms surrounding tissue can influence the efficiency of light‐spectrum harvesting as it determines the light reaching the photosynthetic apparatus (Goessling et al., 2018, 2019).

In general, the species‐specific ability to harvest photons of certain wavelengths is an important ecological trait as phytoplankton species growing under different supplied light spectrum but equal integrated intensities resulted in species specifically different photosynthesis and growth rates (Baba et al., 2012; Jeon et al., 2005; Sánchez‐Saavedra, 2002). This is also acknowledged in biotechnological approaches to optimize phytoplankton culture growth (35/362 articles). Overall, the light cannot be simply seen as a single resource (intensity) for phytoplankton but is acknowledged as a multitude of resources which can differently be exploited (Stomp et al., 2004). Yet, in ecological considerations, the light is often simplified to its intensity, but a more holistic view as a combined light‐climate including information on the available wavelengths is needed and shows an increasing trend in current research (only intensity: 229 articles, medium year of publication: 2004; intensity and spectrum: 133 articles, medium year of publication: 2013).

### 2.2 Changes and variability in light‐climate

In nature, the light‐climate further exhibits a broad temporal variability and is also affected by environmental changes. The terrestrial runoff after strong precipitation which may result in a pulsed “brownification” of coastal waters is, for example, rapidly changing the available intensity and spectrum of light (Thrane et al., 2014). In such a disturbance event, phytoplankton can respond by different mechanisms (Box 1) or will otherwise experience limiting light conditions which can consequently result in a shift in community composition. These response mechanisms present crucial traits for survival as well as optimal usage of variable light conditions and were found to vary strongly between species (Harris et al., 2009).

BOX 1 **Phytoplankton responses to light. Physiological responses are species specific and the timing as well as metabolic cost may decide of competition outcomes in phytoplankton communities. As the terms sensing, photoacclimation, ‐regulation, ‐adaptation, and ‐protection are often used in different contexts, we here state a short definition of those. Especially in older articles the term (photo)adaptation was frequently used to describe acclimation of species, whereas the evolutionary adaptation of species to light was rarely examined.**

*Sensing* of light is mediated by a variety of photoreceptors such as the phytochromes sensing the proportions of spectral wavelength notably the red to far‐red light ratio but also orange, green, and blue wavebands (Rensing et al., 2016; Rockwell et al., 2014). In aquatic environments, where red and far‐red wavelengths strongly attenuate at the surface, phytochromes may act as depth and phytoplankton neighbor sensing mechanism and thus modulate phototaxis (Fortunato et al., 2016). This topic was covered by 11/362 articles.

*Photoacclimation* is the tuning of light harvesting by *de novo* synthesis or degradation of photosynthetic structures to respond to temporary changes to (low‐, high‐, and spectral‐) light conditions (Falkowski & LaRoche, 1991). This mediates the ratio of photosynthetic to photoprotective carotenoids (Brunet et al., 2011) as well as the size of the light antenna of the photosystems (Eberhard et al., 2008; Granata et al., 2019). Further acclimation includes also functional morphological changes (Janssen et al., 2001), for example, within the thylakoid membrane (Lepetit et al., 2012). This topic was covered by 154/362 articles.

*Photoregulation* is the rapidly tuning of the photosynthetic efficiency, for example, by Rubisco activity, photosynthetic state transition, or the xanthophyll cycle without the de novo synthesis or degradation of photosynthetic structures (Raven & Geider, 2003). The phototaxis of mobile phytoplankton species presents another way of photoregulation to available PAR, and diel migration has further been shown to be dependent on the spectral quality (Figueroa et al., 1998). This topic was covered by 24/362 articles.

*Photoadaptation* refers to the evolutionary adaptation of species to long‐term light exposure (Falkowski & LaRoche, 1991). For example, oceanic diatoms were found to be adapted to more constant light conditions by cutback of their photosynthetic apparatus which allows lower iron demands but might have also sacrificed their acclimation abilities to rapid light fluctuations in coastal areas (Lavaud et al., 2007; Strzepek & Harrison, 2004). Additionally, adapted cellular structures can manipulate the intracellular light availability and enhance photosynthesis (Goessling et al., 2018, 2019). This topic was covered by 26/362 articles.

*Photoprotection* includes all reaction types of above if performed to prevent or counteract damaging processes by high (PAR and/or UV)‐light conditions. Structural protection by, for example, diatom frustules, potentially reduces UV radiation (Demmig‐Adams & Adams, 1992; Ellegaard et al., 2016). “Sunscreen” compounds screen against UV radiation (Gao & Garcia‐Pichel, 2011). Excessive radiation energy can be reduced by either heat dissipation by non‐photochemical quenching (NPQ) or chlorophyll fluorescence (Bailey & Grossman, 2008; Demers et al., 1991). Secondary damage is prevented by scavenging of reactive oxygen species using antioxidants (Szymańska et al., 2017). This topic was covered by 49/362 articles.

As for the impacts of light on photosynthesis, the response to a changed light availability was earlier and more often investigated regarding the light intensity without acknowledging responses to the spectrum of light. Especially for the photoacclimation of phytoplankton 101/362 articles were identified, which did not consider acclimation toward a changed light spectrum. This acclimation toward an optimized absorption of the light spectrum or intensity and spectrum in combination was comparably less investigated (53/362 articles). The so‐called complementary chromatic acclimation by changes in the pigment composition was found to be predominantly granted to cyanobacteria being able to increase the proportions of phycoerythrin under green light or phycocyanin under red light within their phycobilisomes to maximize light absorption efficiency (Grossman et al., 1993; Gutu & Kehoe, 2012). In contrast, the complementary chromatic acclimation was tested and observed for a variety of eukaryotic species but for those no general pattern could be determined (Mouget et al., 2004; Vesk & Jeffrey, 1977). In a tradeoff principal, a fast‐responding organism may perform better in frequently changing environments, whereas a slow responding organism may perform better under constant conditions without the perpetual investment in acclimation (see e.g. van Leeuwe et al., 2005). In any case, the acclimation response includes metabolic costs which was found to lower productivity yield at fast changing conditions (Retkute et al., 2015). Especially the protection to high irradiance is important for survival but leads to a decreasing maximum yield in photosynthesis and carbon fixation which is a competitive disadvantage when the irradiance dims (Marra et al., 2000).

Overall, rapid changes in light‐climate are favoring fast acclimating or good endowed species over those who cannot acclimate and efficiently harvest the “new” light‐climate. As a consequence, changes in the light spectrum can change species growth rates (Luimstra et al., 2019) and alter the phytoplankton community composition (Hintz et al., 2021).

### 2.3 Fluctuations in light‐climate

In addition to single changes in the light‐climate, natural irradiance often periodically fluctuates over temporal periods of 10^−8^ s^−1^ up to 10 s^−1^ (Litchman & Klausmeier, 2001). Fast fluctuating underwater light is often given by (1) the formation of waves which lead to rapid refraction effects and focusing of light (Schenck, 1957) and (2) changed positions of phytoplankton during the vertical mixing of a water column, for example, by fast and deep Langmuir circulation (Denman & Gargett, 1983). Longer fluctuations cover (3) meteorological changes as by cloud formation (Nann & Riordan, 1991) as well as global cycles of (4) day–night changes in combination with (5) seasonal changes (seasonal changed solar angle) (Dubinsky, 1986).

For phytoplankton, the experienced light‐climate hence is rather fluctuating than constant in natural systems. Therefore, the response of phytoplankton to fluctuating light is regularly considered in our identified articles (98/362). Due to these fluctuations, phytoplankton might experience on average longer periods under suboptimal conditions (limiting low or inhibiting high light, in regard of the species *P*–*I* curves) (Guislain et al., 2018). If so, growth might be reduced compared to constant conditions even if both conditions have the same daily mean intensity (Köhler et al., 2018). A slow fluctuation would allow a species to acclimate in time to efficiently perform under the changed conditions, whereas the resource use of an individual cannot be efficient if the resource fluctuates faster than acclimation is feasible (Cullen & Lewis, 1988; Koussoroplis et al., 2017). Contrariwise, very fast fluctuations (frequencies >1 s^−1^) in light supply were found to enhance photosynthesis (Grobbelaar et al., 1996; Walsh & Legendre, 1983) which is also considered in biotechnological approaches (21/362 articles) as it reduces energy costs in production (Abu‐Ghosh et al., 2016). Possible explanations for that are the match of the photon input rate to photosynthesis (e.g., electron transfer rates), or reduced photoinhibition (Abu‐Ghosh et al., 2016). As described above, the species‐specific acclimation to (not necessarily recurring) changes in light takes time, costs energy, and is limited to the degree of plasticity which applies for the response to fluctuation, too (van Leeuwe et al., 2005; Nicklisch, 1998).

The species‐specific response to light fluctuations can further lead to changes in the phytoplankton community compositions and diversity (Flöder et al., 2002; Guislain et al., 2018). Litchman and Klausmeier (2001) found that the fluctuating light generally promotes opportunistic—often fast growing—species but on the other side slows or even prevent competitive exclusion, thus allows a higher species richness. Thereby, coexistence of species is favored if they differ strongly in the gleaner‐opportunistic tradeoff which applies to competition between a species that performs well in the low‐light intensity phase due to a low required intensity at its compensation point (*I*_c_), while a species with a high maximum photosynthesis or growth rate (*P*_max_) performs better in the high‐light intensity phase.

In contrast to atmospheric or planetary reasons of light fluctuation, changes of the position within the water column do not only affect the light intensity, but also the spectrum as experienced by phytoplankton. In this regard, we identified 38/362 articles which investigated the effects of vertical mixing of the water column. On the one hand, the low‐light availability at a large mixing depth negatively affects phytoplankton growth (Bernhardt et al., 2008). On the other hand, the mixing also reduces the time spent at the surface and therefore potentially mitigates photoinhibition, and enhances photosynthesis, resulting in higher growth compared to static light environments (Marra, 1978). Additionally, the mixing counteracts sinking losses of phytoplankton and total phytoplankton biomass was found to be highest at intermediate mixing depths (Diehl et al., 2002).

As for the light fluctuation alone, some species are more adapted to static intensities while others are more competitive under well‐mixed conditions and the vertical mixing can therefore affect community composition according to the photosynthesis traits of the included species (Litchman, 2008; Strzepek & Harrison, 2004). In addition, this is not only due to photosynthetic traits as Huisman et al. (2004) found that a weak mixing favors buoyant cyanobacteria over fast sinking diatoms and green algae as the former can float upwards and shade the latter. Indirectly, the vertical mixing may also resuspend particles at shallow waters which lowers the overall light availability (Helbling et al., 2015). But the suspended sediment can mitigate photoinhibition effects which was also found to lead to increased productivity as compared to static environments (Mallin & Paerl, 1992).

Overall, the consequences of fluctuating light are dependent on the timescales and amplitude as well as the individual species traits and other environmental factors. Therefore, a generalization of the response for fluctuating light is difficult. We highlight the need for more investigations of variability of light and fluctuations as those are more common in nature than static (often simplified laboratory) conditions.

## 3 COMPETITION FOR LIGHT AND VERTICAL ARRANGEMENT OF PHYTOPLANKTON IN (NON‐STATIC) LIGHT GRADIENTS

As highlighted above, the different species‐specific requirements for light allow for a niche separation along the water column's light gradient. The individual pigment composition enables complementary light utilization as different parts of the light spectrum can be exploited (Glover et al., 1987; Ting et al., 2002) and promotes biodiversity (Stomp et al., 2004; Striebel et al., 2009), whereas a curtailed spectrum leads to competition and selection (Luimstra et al., 2019; Rocap et al., 2003; Stomp, Huisman, Voros, et al., 2007).

Vertical arrangement of phytoplankton species along the poorly mixed water column's light gradient can be of key importance in this regard and was considered in 15/59 articles. The respective light spectrum at certain depths within a water column predicts competition outcomes and subsequently the composition of local phytoplankton communities. This concept was very early stated by Engelmann (1883) and supported by few observations (Hickman et al., 2009; Holtrop et al., 2021; Stomp, Huisman, Stal, et al., 2007). However, it does not necessarily hold predictable for highly variable, that is, well‐mixed environments (Jäger et al., 2008) and when additionally considering the availability of other (co‐)limiting resources, such as nutrients (Mellard et al., 2012). In addition, phytoplankton growth at the surface decreases light intensity due self‐shading and thus feeds back into the light availability (Shigesada & Okubo, 1981). This does not only affect the intensity of light but also its spectrum, as the absorption is wavelength specific. This biological light filtering, that is, selective absorption of wavelengths passing phytoplankton in the upper water layer shifts the spectrum in the lower water column selectively favors those who can efficiently use the remaining light (Montesinos et al., 1983). The concept of “luxury consumption,” the excess consumption of a non‐limiting resource (Chapin et al., 1990), has also recently been discussed with regard to phytoplankton (Luimstra et al., 2019). Cyanobacteria with phycobilisomes are less effective in utilizing blue light than eukaryotic phytoplankton, although potentially absorbing it to a similar extent, making it unavailable to cells below (de Mazancourt & Schwartz, 2012). This applies also for light which is not used for photosynthesis but absorbed by cell tissue. Harris et al. (2009) found that the photoprotective measures of phytoplankton such as the synthesis of “sunscreen” substances can further increase the potential shading effect. Such photoacclimation and ‐protection measures by pigment adjustment would alter the absorption spectra and respectively feed back to the transmitted light‐spectrum behind the phytoplankton cell. Hypothetically, in a steady water column, this could affect organisms beneath in a cascade sequence of several species acclimating to the available light at their depth and respective remaining light spectrum. However, we could not identify such studies which combines the effects of species‐specific light absorption and acclimation to the available spectrum on the transmitted/remaining light spectrum available for other species in a water column. This topic becomes even more complex as it also implies that phototaxis by certain species not only optimizes access to light but further actively affects the shading of competitors.

Overall, the vertical arrangement of cells according to the ambient light‐intensity and ‐spectrum is an intricate combination of the physical environment and competing species with potential for further investigations.

## 4 ECOLOGICAL EFFECTS OF LIGHT‐CLIMATE CHANGES ON PHYTOPLANKTON UNDER FUTURE PREDICTIONS OF CLIMATIC CHANGE

**FIGURE 2:**
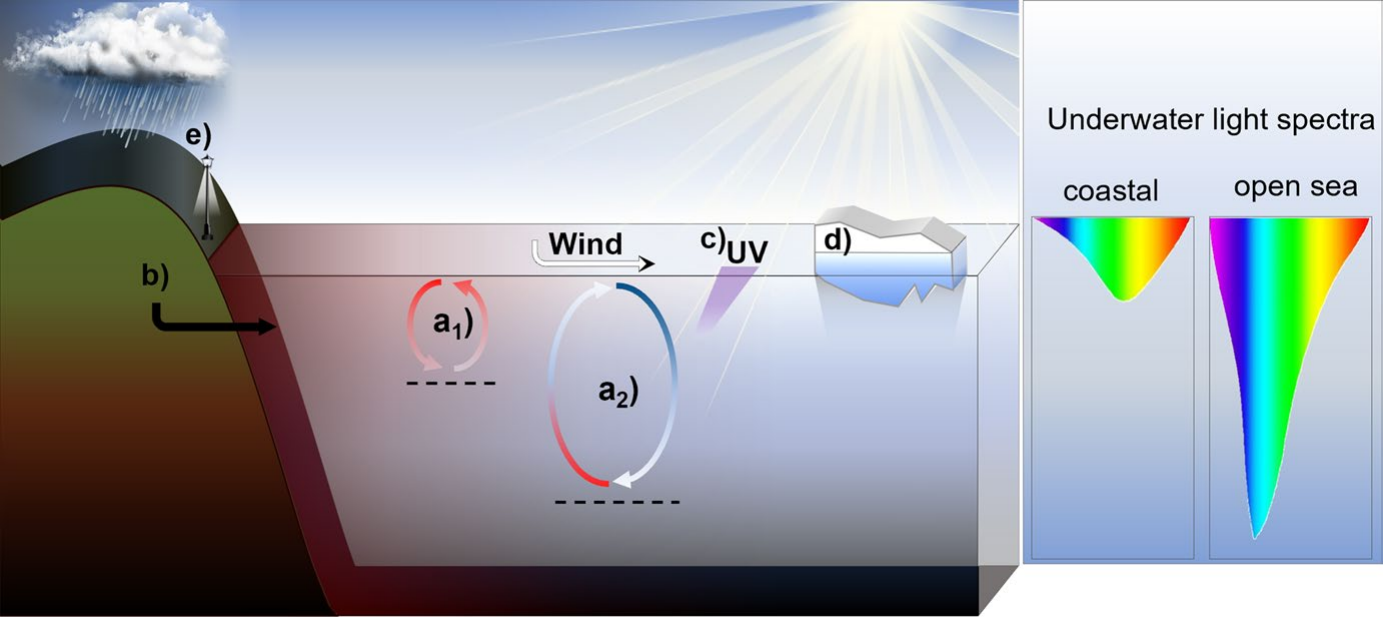
Schematic overview of climate change effects on the underwater light as experienced by phytoplankton. Fundamentally, the incident sunlight decreases with water depth and shapes spectral niches (for a detailed niche formation, see Holtrop et al., 2021; Stomp, Huisman, Stal, et al., 2007). (a) Wind and temperature changes will affect stratification of the water column as well as mixing depth which affects light availability for mixed phytoplankton. (a_1_) Increasing surface temperatures may increase thermal stratification and reduces mixing depth. (a_2_) If exposed to surface winds, those can cool down surface waters and destabilize stratification which allows a deeper mixing depth. Mixing arrow colors roughly illustrate the water temperature at respective depth (red: warmer surface water in scenario a_1_), blue: colder surface water in a_2_). (b) Increasing cDOM subsidies in coastal areas are expected due to stronger precipitation and agricultural land use. The input of cDOM shades the overall light availability and light spectrum. Conversely, cDOM might protect phytoplankton due to shading of UV light. (c) UV light, due to ozone depletion rapidly attenuates below the water surface, can damage phytoplankton and degrades cDOM. (d) Sea ice covers the water surface and reflects sunlight (albedo). Melting will expose the surface to wind and light. (e) Artificial light at night induces light pollution in a close‐by environment with light levels potentially exceeding the lower limit of photosynthesis

Various environmental changes, such as elevated CO_2_, elevated temperature as well as reductions in ice and snow coverage are expected to directly affect phytoplankton communities (Hays et al., 2005) and are of high importance when investigating ecosystem functionality (Isbell et al., 2011). The direct implications of an indirectly changing underwater light‐climate are partially neglected, and we aim to structure their ecological consequences (Figure 2). Despite the thematic partition hereinafter, these changes cannot be seen as isolated factors as they interact with each other and act simultaneously on phytoplankton (Häder et al., 2014). For this objective, we identified 255 articles covering light‐climate change effects on phytoplankton by altered stratification and vertical mixing, lake and coastal darkening, UV radiation, melting sea ice, and ecological light pollution.

### 4.1 Ocean and lake stratification as well as changing mixing conditions

The effects of light due to stratification and altered vertical mixing patterns, as potential consequence of climate change, on phytoplankton were covered in 89/255 of the identified articles. Thereby, stratification of large waterbodies does not only limit nutrient availability in the epilimnion, but further holds phytoplankton at depths with rather constant light conditions (Figure 2a). Increasing average temperatures as well as wind sheering is predicted to change stratification onset, depth, and stability which is overall correlated to net primary production (Behrenfeld et al., 2006; Berger et al., 2010; Wahl & Peeters, 2014). In lakes, surrounded by trees with low winds, surface waters will warm up, leading to stronger differences in densities among water layers, and consequently a lower probability for deep mixing events disturbing the stratification between the epi‐ and hypolimnion (Figure 2a1). On the other hand, when local winds chill surface temperatures, the stratification thermocline will become destabilized and allows for deeper vertical mixing (Saros et al., 2012) (Figure 2a2). Additionally, in upwelling regions, winds are overall expected to increase and favor upwelling as well as suppresses stratification (Sydeman et al., 2014). This has implications for the mixing layer depth but was found to vary regionally as well as seasonally (Somavilla et al., 2017). When being dragged through different light regimes in interaction with variable nutrient supply dependent on the mixing depth, phytoplankton communities are expected to change in their species composition (Marzetz et al., 2020; Saros et al., 2012). Thus, increased vertical mixing depth acts selectively by limiting light in spectrum and intensity at depth and reduces phytoplankton growth due to longer periods at depth (Lehman, 2002; Northington et al., 2019). On the other hand, when the mixing depth or the water column itself is shallow enough for light being not limited, the higher availability of nutrients from hypolimnic water and sediments would promote phytoplankton growth (Planas & Paquet, 2016). Contrariwise, when primary producers are trapped within the epilimnion by strong stratification, the broader PAR bandwidth availability may facilitate higher diversity as it can be used concertedly (Striebel et al., 2009). This, however, might then be again mitigated if nutrients are limiting or the impact of damaging UV radiation gains influence (Häder et al., 2014).

Based on these scenarios, we expect future outcomes to be spatially high variable and be an individual combination of multiple environmental factors. The effects of altered light fluctuation by predicted vertical mixing can also be counteracted by other climate changes such as increasing acidification and nutrient inputs (Bermejo et al., 2020), which complicates predictions but opens directions for further research and combined approaches.

### 4.2 Lake and coastal ocean darkening

Increasing storm events, precipitation, melting glaciers, and thawing permafrost, which are expected due to climate change (de Wit et al., 2016; Grosse et al., 2011; Parry et al., 2007; Weyhenmeyer et al., 2015) but also increasing land use and urbanization (Lyu et al., 2021) can lead to terrestrial runoffs into adjacent waters (Vizzo et al., 2021; Weyhenmeyer & Karlsson, 2009). By this, the input of cDOM to small water bodies as well as coastal shores (Figure 2b) affects the underwater light in terms of increasing “brownification” and overall darkening (Dutkiewicz et al., 2019; Roulet & Moore, 2006; Thrane et al., 2014). Storm events may raise sediment in shallow lakes which increases fluxes of nutrients from the sediment as well as water turbidity (Beaver et al., 2013; Blom et al., 1994). On the one hand, a darker waterbody increases also in its heat absorption at the surface, leading to a potential increase in thermal stratification (Williamson et al., 2019). On the other hand, Houser (2006) identified lower temperatures but higher ranges in daily temperature changes in colored compared to clear lakes. The authors argue that stronger light absorption by cDOM could reduce heat storage in the hypolimnion and effects of watercolor on water temperature are also depending on groundwater exchange.

The ecological effects of “brownification” and darkening on phytoplankton as consequence of climate change were investigated in 85/255 articles. As a general consequence, the light limitation by increasing levels of cDOM is expected to reduce phytoplankton growth and shift community composition, but at medium cDOM concentrations the additional nutrients contrariwise can support growth (Feuchtmayr et al., 2019; Thrane et al., 2014; Villafane et al., 2018). This antagonism of light and nutrient availability becomes apparent if the decrease in light availability leads to reduction of benthic phytoplankton. As the benthic phytoplankton is intercepting arising nutrients from the sediments, this would then result in more nutrients reaching the water surface, which, in turn, promotes the growth of pelagic primary producers and leads to even more shading (Vasconcelos et al., 2016). The acclimation of cells to low‐light conditions was additionally found to adversely result in a higher susceptibility to UV radiation (Helbling et al., 2013). Despite the high attenuation of UV light by cDOM (Gibson et al., 2000) (see also below), low‐light acclimated cells can be rapidly exposed to high levels of UV radiation if cDOM dissipates, the cells are dragged to the direct water surface, or the light becomes focused by waves (Schubert et al., 2001).

In general, regions where cDOM inputs and wind stress are increasing are therefore predicted to be reduced in phytoplankton growth (Helbling et al., 2015). Among the identified studies, knowledge gaps arise due to the high variability in cDOM composition and degradation (Hansen et al., 2016) and hence wavelength‐specific light attenuation on variable timescales.

### 4.3 UV radiation impact

Previous anthropogenic impacts reduced the atmospheric ozone layer which led to increasing UV radiation within aquatic ecosystems with variable but damaging consequences for its inhabitants (Smith, 1989; Williamson et al., 2014, 2019). Due to the Montreal Protocol, stratospheric ozone depletion could successfully be cushioned by reduction of damaging chlorofluorocarbons but to date the ozone layer has not recovered and is continually affected by climate change (Bais et al., 2015; Williamson et al., 2019). Within this context, the effects of UV radiation on phytoplankton (Figure 2c) are important and investigated in 78/255 articles.

In combination with stratification, the depth of the epilimnion would either strengthen the exposure of phytoplankton to UV radiation if restricted to upper layers (Häder et al., 2011) or, if deep enough, allow avoidance from UV radiation (Helbling et al., 2013) and allows for recovery of the photosynthetic apparatus after inhibition (Smyth et al., 2017). Therefore, the interaction of stratification and mixing depth with UV radiation strongly depend on the pace and mixing depth (Neale et al., 1998). The penetration depth of UV‐light is further directly related to the concentration of cDOM due to its absorption (Gibson et al., 2000; Harrison & Smith, 2009). Fluorescent dissolved organic matter (fDOM) and cDOM strongly absorb and attenuates UV light but, in turn, degrades (Hansen et al., 2016; Miranda et al., 2018). This degradation allows a deeper UV penetration and permits UV damaging effects (Williamson et al., 2014) but at the same time increases the availability of PAR (Schubert et al., 2001).

Where primary producers cannot avoid the UV radiation by active movement, this gains a high ecological relevance. Especially sessile organisms such as endosymbiotic zooxanthellae of corals are recognized to become photo‐inhibited which leads to coral bleaching under additionally rising temperature (Hoegh‐Guldberg, 1999). Phytoplankton at the surface of waters is in principle directly negatively affected by damaging UV radiation (Harrison & Smith, 2009). The effects of UV radiation on phytoplankton are species specific and linked to other consequences of climate change as well as other environmental factors, such as nutrient availability and thermal stress (Harrison & Smith, 2009; Jin et al., 2019; Williamson et al., 2019). The tolerance and protection against UV radiation displays a strong advantage and may favor protected species (Häder et al., 2011) and species living in niche environments of high UV radiation (Wu et al., 2017). The other way around, the pigment group of phycobilins are highly sensitive to UV radiation making it a disadvantage to cyanobacteria, red algae, glaucophytes, and cryptomonads (Häder & Gao, 2015). And due to ocean acidification, calcareous coccolithophorids are becoming more susceptible to UV radiation because of shell thinning (Gao & Häder, 2017). However, general trends of UV radiation on phytoplankton community composition are hardly to generalize as only a limited number of comparison of species UV susceptibility was made (Harrison & Smith, 2009). The UV radiation availability and its effects are temporally variable, which might be considered as a temporary disturbance on a phytoplankton community. Such as the PAR displays a multitude of resources, the damaging effects of UV radiation are wavelength dependent as well, with shorter wavelengths (UV‐B, 280–315 nm) having generally stronger damaging potential (Peng et al., 2017) but longer wavelengths (UV‐A, 315–400 nm) penetrating the water column deeper. In contrast to damaging UV‐B, UV‐A radiation was found to promote photosynthetic carbon fixation under low or fluctuating irradiance (Beardall et al., 2014; Gao et al., 2007). 34/78 of the identified studies did not distinguish between effects of the UV subtypes UV‐A and ‐B and 16/78 focused only on UV‐B; thus, adverse effects may be over‐estimated when projecting to natural environments where short wavelengths are attenuated the strongest (Williamson et al., 2019). Williamson et al. (2019) further highlight the lack of data for spectral dependence of UV radiation effects as experiments are often conducted under artificial UV light sources being not as complex as sunlight and further point out to consider the interactions of other climate change effects with UV radiation.

### 4.4 Melting sea ice

The trend of global warming drives melting and reduction of sea ice as well as snow coverage (Lannuzel et al., 2020; Magnuson et al., 2000). Among multiple effects, this ice thinning primarily reduces light attenuation and receding of ice cover lowers albedo. Within this context, we identified 32/255 articles covering the effects of changed light by melting sea ice (Figure 2d). The trends of increasing in PAR and temperature are expected to enhance productivity and can cause earlier seasonal phytoplankton bloom onsets (Gronchi et al., 2021; Patara et al., 2012). Subsequently, enhanced growth of phytoplankton darkens the surface of ice and water, leading to a higher heat absorption resulting in an ice melting feedback (Williamson et al., 2020). Contrariwise, the sudden exposure to full incident light can result in photoinhibition of phytoplankton (Kauko et al., 2017). The outcome of increased PAR on the one side but photoinhibition on the other side thereby depends not only on the intensity of light, but also on the species adaption and acclimation mechanisms (Croteau et al., 2021; Juhl & Krembs, 2010). Additionally to PAR availability, the UV radiation was found to presumably increase up to 10 times in arctic surface water which also negatively affects surface phytoplankton (Fountoulakis et al., 2014). Rapid melting events but also the duration and intensity of ice coverage thus can change community composition (Lenard & Wojciechowska, 2013; Williamson et al., 2020).

To better predict future outcomes of sea ice melting, Lannuzel et al. (2020) and Steiner et al. (2015) emphasized the need of new and sustained field data, longer time series as well as improved models of environmental changes, but also further insights on biological mechanisms and processes such as phytoplankton individual responses, community compositions, and trophic interactions.

### 4.5 Light pollution

In comparison to sunlight, artificial light pollution (Figure 2e) does only play a minor role in terms of resource for primary producers due to its restricted areas and our search identified 4/255 articles. Yet, artificial light at night (ALAN) in coastal areas can penetrate the whole water column and affect ecosystems (Davies et al., 2020). In shore near waters, the illumination, even though being of comparable low intensity and locally restricted, was found to exceed the lower limit for photosynthesis when in combination with full moon light (Raven & Cockell, 2006). Additionally, caves which are lit for touristic purposes are known to exhibit the so‐called “Lampenflora” consisting of moss and algae. Thus, trophic processes in a total light excluded microbial ecosystem became severely changed (Popkova et al., 2019). However, except for those few specific examples, general effects of light pollution in terms of being ecological resources are insufficiently investigated or focused on animals (‐behavior) or terrestrial systems (Gaston et al., 2013).

## 5 DISCUSSION OF THE SYSTEMATIC LITERATURE MAP

In this study, we mapped the conducted research on ecological consequences of light in terms of intensity, spectrum, variability, and aspects of predicted climate change to extend existing reviews about light effects on phytoplankton. However, this mapping approach has some restrictions. (1) The wide field of research in photosynthesis and primary production forced us to define the literature search in a specific way, thus including restriction within the fields of science. By that, the declaration of numbers of identified studies is only as robust as the search itself. Yet, our selection of search terms resulted in a high number of identified records and thus a well‐appointed overview which was additionally amended with studies known to the authors. (2) Even though we aimed to cover direct light effects alone, most research included (inseparably) coupled effects as well, meaning that effects of light on phytoplankton are often coupled to and investigated with effects of, for example, nutrient availability. (3) We aimed only at direct effects of light on phytoplankton, but also want to highlight the need for investigations on indirect effects within food webs such as reduced grazing pressure due to UV damage on zooplankton (Williamson et al., 2019). Therefore, a generalization of the herein mapped consequences is difficult and should be considered as motivation for further research.

## 6 CONCLUSION

By systematically mapping the published research, we described and structured the ecological consequences of photosynthetic light harvesting in aquatic environments. We highlighted light as highly variable and as a multitude of resources and explained competition and possible coexistence of photosynthetic species which shapes communities and succession. Undisputed is the effect of light intensity and spectrum on the ecology of phytoplankton communities but consequences may differ strongly between ecosystems. Future alterations in the underwater light availability and spectrum are an indirect consequence of anthropogenic climate change and will certainly alter primary producer's community compositions and ecological interactions. We highly encourage further research in the discussed topics and the consideration of the variability of light in both spectrum and intensity. Together with progress in climate change research, this will help to improve prediction of consequences for phytoplankton communities.

## CONFLICT OF INTEREST

The authors declare no conflict of interest.

## AUTHOR CONTRIBUTIONS

**Nils Hendrik Hintz:** Conceptualization (equal); Data curation (equal); Formal analysis (equal); Investigation (equal); Validation (equal); Visualization (equal); Writing – original draft (equal); Writing – review & editing (equal). **Brian Schulze:** Conceptualization (equal); Investigation (equal); Writing – review & editing (equal). **Alexander Wacker:** Conceptualization (equal); Funding acquisition (equal); Investigation (equal); Supervision (equal); Writing – review & editing (equal). **Maren Striebel:** Conceptualization (equal); Funding acquisition (equal); Investigation (equal); Supervision (equal); Writing – original draft (equal); Writing – review & editing (equal).

### OPEN RESEARCH BADGES

This article has been awarded Open Materials, Open Data, Preregistered Research Designs Badges. Preregistration was done via the Open Science Framework at https://osf.io/ky3ut. All materials and data are publicly accessible via the Supporting Information and via Dryad at (https://doi.org/10.5061/dryad.7h44j0zw5).

## Supporting information

Appendix S1

Appendix S2

Appendix S3

## Data Availability

Additional methods (search terms, flowchart, checklist) are available as Supporting Information. The full reference list of analyzed literature is accessible on Dryad (https://doi.org/10.5061/dryad.7h44j0zw5).

## References

[ece38753-bib-0001] Abu‐Ghosh, S. , Fixler, D. , Dubinsky, Z. , & Iluz, D. (2016). Flashing light in microalgae biotechnology. Bioresource Technology, 203, 357–363. 10.1016/j.biortech.2015.12.057 26747205

[ece38753-bib-0002] Baba, M. , Kikuta, F. , Suzuki, I. , Watanabe, M. M. , & Shiraiwa, Y. (2012). Wavelength specificity of growth, photosynthesis, and hydrocarbon production in the oil‐producing green alga Botryococcus braunii. Bioresource Technology, 109, 266–270. 10.1016/j.biortech.2011.05.059 21683581

[ece38753-bib-0003] Bailey, S. , & Grossman, A. (2008). Photoprotection in Cyanobacteria: Regulation of light harvesting. Photochemistry and Photobiology, 84, 1410–1420. 10.1111/j.1751-1097.2008.00453.x 19067963

[ece38753-bib-0004] Bais, A. F. , McKenzie, R. L. , Bernhard, G. , Aucamp, P. J. , Ilyas, M. , Madronich, S. , & Tourpali, K. (2015). Ozone depletion and climate change: Impacts on UV radiation. Photochemical & Photobiological Sciences, 14, 19–52. 10.1039/c4pp90032d 25380284

[ece38753-bib-0005] Beardall, J. , Stojkovic, S. , & Gao, K. S. (2014). Interactive effects of nutrient supply and other environmental factors on the sensitivity of marine primary producers to ultraviolet radiation: implications for the impacts of global change. Aquatic Biology, 22, 5–23. 10.3354/ab00582

[ece38753-bib-0006] Beaver, J. R. , Casamatta, D. A. , East, T. L. , Havens, K. E. , Rodusky, A. J. , James, R. T. , Tausz, C. E. , & Buccier, K. M. (2013). Extreme weather events influence the phytoplankton community structure in a large lowland subtropical lake (Lake Okeechobee, Florida, USA). Hydrobiologia, 709, 213–226. 10.1007/s10750-013-1451-7

[ece38753-bib-0007] Behrenfeld, M. J. , O'Malley, R. T. , Siegel, D. A. , McClain, C. R. , Sarmiento, J. L. , Feldman, G. C. , Milligan, A. J. , Falkowski, P. G. , Letelier, R. M. , & Boss, E. S. (2006). Climate‐driven trends in contemporary ocean productivity. Nature, 444, 752–755. 10.1038/nature05317 17151666

[ece38753-bib-0008] Berger, S. A. , Diehl, S. , Stibor, H. , Trommer, G. , & Ruhenstroth, M. (2010). Water temperature and stratification depth independently shift cardinal events during plankton spring succession. Global Change Biology, 16, 1954–1965. 10.1111/j.1365-2486.2009.02134.x

[ece38753-bib-0009] Bermejo, P. , Duran‐Romero, C. , Villafane, V. E. , & Helbling, E. W. (2020). Influence of fluctuating irradiance on photosynthesis, growth and community structure of estuarine phytoplankton under increased nutrients and acidification. Journal of Experimental Marine Biology and Ecology, 526. 10.1016/j.jembe.2020.151348

[ece38753-bib-0010] Bernhardt, J. , Elliott, J. A. , & Jones, I. D. (2008). Modelling the effects on phytoplankton communities of changing mixed depth and background extinction coefficient on three contrasting lakes in the English Lake District. Freshwater Biology, 53, 2573–2586. 10.1111/j.1365-2427.2008.02083.x

[ece38753-bib-0011] Blom, G. , Vanduin, E. H. S. , & Lijklema, L. (1994). Sediment resuspension and light conditions in some shallow Dutch Lakes. Water Science and Technology, 30, 243–252. 10.2166/wst.1994.0534

[ece38753-bib-0012] Brunet, C. , Johnsen, G. , Lavaud, J. , & Roy, S. (2011). Pigments and photoacclimation processes. In S. Roy , C. Llewellyn , E. Egeland , & G. Johnsen (Eds.), Phytoplankton Pigments: Characterization, Chemotaxonomy and Applications in Oceanography. Cambridge Environmental Chemistry Series. (pp. 445–471). Cambridge University Press. 10.1017/CBO9780511732263.017

[ece38753-bib-0013] Chapin, F. S. , Schulze, E. D. , & Mooney, H. A. (1990). The ecology and economics of storage in plants. Annual Review of Ecology and Systematics, 21, 423–447. 10.1146/annurev.ecolsys.21.1.423

[ece38753-bib-0014] Coble, P. G. (2007). Marine optical biogeochemistry: the chemistry of ocean color. Chemical Reviews, 107, 402–418. 10.1021/cr050350+ 17256912

[ece38753-bib-0015] Croteau, D. , Guerin, S. , Bruyant, F. , Ferland, J. , Campbell, D. A. , Babin, M. , & Lavaud, J. (2021). Contrasting nonphotochemical quenching patterns under high light and darkness aligns with light niche occupancy in Arctic diatoms. Limnology and Oceanography, 66, S231–S245. 10.1002/lno.11587

[ece38753-bib-0016] Cullen, J. J. , & Lewis, M. R. (1988). The kinetics of algal photoadaptation in the context of vertical mixing. Journal of Plankton Research, 10, 1039–1063. 10.1093/plankt/10.5.1039

[ece38753-bib-0017] Davies, T. W. , McKee, D. , Fishwick, J. , Tidau, S. , & Smyth, T. (2020). Biologically important artificial light at night on the seafloor. Scientific Reports, 10, 12545. 10.1038/s41598-020-69461-6 32719492 PMC7385152

[ece38753-bib-0018] de Mazancourt, C. , & Schwartz, M. W. (2012). Starve a competitor: Evolution of luxury consumption as a competitive strategy. Theoretical Ecology, 5, 37–49. 10.1007/s12080-010-0094-9

[ece38753-bib-0019] de Wit, H. A. , Valinia, S. , Weyhenmeyer, G. A. , Futter, M. N. , Kortelainen, P. , Austnes, K. , Hessen, D. O. , Räike, A. , Laudon, H. , & Vuorenmaa, J. (2016). Current browning of surface waters will be further promoted by wetter climate. Environmental Science & Technology Letters, 3, 430–435. 10.1021/acs.estlett.6b00396

[ece38753-bib-0020] Demers, S. , Roy, S. , Gagnon, R. , & Vignault, C. (1991). Rapid light‐induced‐changes in cell fluorescence and in xanthophyll‐cycle pigments of Alexandrium‐Excavatum (Dinophyceae) and Thalassiosira‐Pseudonana (Bacillariophyceae) – A photo‐protection mechanism. Marine Ecology Progress Series, 76, 185–193. 10.3354/meps076185

[ece38753-bib-0021] Demmig‐Adams, B. , & Adams, W. W. (1992). Photoprotection and other responses of plants to high light stress. Annual Review of Plant Physiology and Plant Molecular Biology, 43, 599–626. 10.1146/annurev.pp.43.060192.003123

[ece38753-bib-0022] Denman, K. L. , & Gargett, A. E. (1983). Time and space scales of vertical mixing and advection of phytoplankton in the upper ocean. Limnology and Oceanography, 28, 801–815. 10.4319/lo.1983.28.5.0801

[ece38753-bib-0023] Diehl, S. , Berger, S. , Ptacnik, R. , & Wild, A. (2002). Phytoplankton, light, and nutrients in a gradient of mixing depths: Field experiments. Ecology, 83, 399–411. 10.2307/2680023

[ece38753-bib-0024] Dokulil, M. T. , & Kaiblinger, C. (2009). Phytoplankton productivity. In G. E. Likens (Ed.), Encyclopedia of inland waters (pp. 210–218). Academic Press. 10.1016/B978-012370626-3.00140-X

[ece38753-bib-0025] Dubinsky, Z. (1986). Productivity of algae under natural conditions. In A. Richmond (Ed.), Handbook of microalgal mass culture (1st ed.). CRC Press. 10.1201/9780203712405

[ece38753-bib-0026] Dutkiewicz, S. , Hickman, A. E. , Jahn, O. , Henson, S. , Beaulieu, C. , & Monier, E. (2019). Ocean colour signature of climate change. Nature Communications, 10, 578. 10.1038/s41467-019-08457-x PMC6362115 30718491

[ece38753-bib-0027] Eberhard, S. , Finazzi, G. , & Wollman, F. A. (2008). The dynamics of photosynthesis. Annual Review of Genetics, 42, 463–515. 10.1146/annurev.genet.42.110807.091452 18983262

[ece38753-bib-0028] Edwards, K. F. , Thomas, M. K. , Klausmeier, C. A. , & Litchman, E. (2015). Light and growth in marine phytoplankton: allometric, taxonomic, and environmental variation. Limnology and Oceanography, 60, 540–552. 10.1002/lno.10033

[ece38753-bib-0029] Ellegaard, M. , Lenau, T. , Lundholm, N. , Maibohm, C. , Friis, S. M. M. , Rottwitt, K. , & Su, Y. (2016). The fascinating diatom frustule—can it play a role for attenuation of UV radiation? Journal of Applied Phycology, 28, 3295–3306. 10.1007/s10811-016-0893-5

[ece38753-bib-0030] Engelmann, T. W. (1883). Farbe und assimilation. Botanische Zeitung, Jahrg. 41, 1–13, 17–29. https://www.biodiversitylibrary.org/item/104958

[ece38753-bib-0031] Falkowski, P. G. (1980). Light‐shade adaptation in marine phytoplankton primary productivity in the sea. Springer. 10.1038/483S17a PMC440685 16661484

[ece38753-bib-0032] Falkowski, P. (2012). Ocean science: The power of plankton. Nature, 483, S17–S20. 10.1038/483S17a 22378122

[ece38753-bib-0033] Falkowski, P. G. , & Laroche, J. (1991). Acclimation to spectral irradiance in algae. Journal of Phycology, 27, 8–14. 10.1111/j.0022-3646.1991.00008.x

[ece38753-bib-0034] Falkowski, P. G. , & Owens, T. G. (1978). Effects of light‐intensity on photosynthesis and dark respiration in 6 species of marine‐phytoplankton. Marine Biology, 45, 289–295. 10.1007/bf00391815

[ece38753-bib-0035] Feuchtmayr, H. , Pottinger, T. G. , Moore, A. , de Ville, M. M. , Caillouet, L. , Carter, H. T. , Pereira, M. G. , & Maberly, S. C. (2019). Effects of brownification and warming on algal blooms, metabolism and higher trophic levels in productive shallow lake mesocosms. Science of the Total Environment, 678, 227–238. 10.1016/j.scitotenv.2019.04.105 31075590

[ece38753-bib-0036] Field, C. B. , Behrenfeld, M. J. , Randerson, J. T. , & Falkowski, P. (1998). Primary production of the biosphere: integrating terrestrial and oceanic components. Science, 281, 237–240. 10.1126/science.281.5374.237 9657713

[ece38753-bib-0037] Figueroa, F. L. , Niell, F. X. , Figueiras, F. G. , & Villarino, M. L. (1998). Diel migration of phytoplankton and spectral light field in the Ría de Vigo (NW Spain). Marine Biology, 130, 491–499. 10.1007/s002270050269

[ece38753-bib-0038] Flöder, S. , Urabe, J. , & Kawabata, Z. (2002). The influence of fluctuating light intensities on species composition and diversity of natural phytoplankton communities. Oecologia, 133, 395–401. 10.1007/s00442-002-1048-8 28466210

[ece38753-bib-0039] Fortunato, A. E. , Jaubert, M. , Enomoto, G. , Bouly, J. P. , Raniello, R. , Thaler, M. , Malviya, S. , Bernardes, J. S. , Rappaport, F. , Gentili, B. , Huysman, M. J. , Carbone, A. , Bowler, C. , D'Alcala, M. R. , Ikeuchi, M. , & Falciatore, A. (2016). Diatom phytochromes reveal the existence of far‐red‐light‐based sensing in the ocean. The Plant Cell, 28, 616–628. 10.1105/tpc.15.00928 26941092 PMC4826011

[ece38753-bib-0040] Fountoulakis, I. , Bais, A. F. , Tourpali, K. , Fragkos, K. , & Misios, S. (2014). Projected changes in solar UV radiation in the Arctic and sub‐Arctic Oceans: Effects from changes in reflectivity, ice transmittance, clouds, and ozone. Journal of Geophysical Research‐Atmospheres, 119, 8073–8090. 10.1002/2014jd021918

[ece38753-bib-0041] Gao, K. , & Häder, D.‐P. (2017). Effects of ocean acidification and UV radiation on marine photosynthetic carbon fixation. In Systems biology of marine ecosystems. 10.1007/978-3-319-62094-7_12

[ece38753-bib-0042] Gao, K. , Wu, Y. , Li, G. , Wu, H. , Villafane, V. E. , & Helbling, E. W. (2007). Solar UV radiation drives CO2 fixation in marine phytoplankton: a double‐edged sword. Plant Physiology, 144, 54–59. 10.1104/pp.107.098491 17494919 PMC1913777

[ece38753-bib-0043] Gao, Q. , & Garcia‐Pichel, F. (2011). Microbial ultraviolet sunscreens. Nature Reviews Microbiology, 9, 791–802. 10.1038/nrmicro2649 21963801

[ece38753-bib-0044] Gaston, K. J. , Bennie, J. , Davies, T. W. , & Hopkins, J. (2013). The ecological impacts of nighttime light pollution: A mechanistic appraisal. Biological Reviews of the Cambridge Philosophical Society, 88, 912–927. 10.1111/brv.12036 23565807

[ece38753-bib-0045] Gibson, J. A. E. , Vincent, W. F. , Nieke, B. , & Pienitz, R. (2000). Control of biological exposure to UV radiation in the Arctic Ocean: Comparison of the roles of ozone and riverine dissolved organic matter. Arctic, 53, 372–382. 10.14430/arctic868

[ece38753-bib-0046] Glazer, A. N. (1985). Light harvesting by phycobilisomes. Annual Review of Biophysics and Biophysical Chemistry, 14, 47–77. https://doi.org/10.1146%2Fannurev.bb.14.060185.000403 10.1146/annurev.bb.14.060185.000403 3924069

[ece38753-bib-0047] Glover, H. E. , Keller, M. D. , & Spinrad, R. W. (1987). The effects of light quality and intensity on photosynthesis and growth of marine eukaryotic and prokaryotic phytoplankton clones. Journal of Experimental Marine Biology and Ecology, 105, 137–159. 10.1016/0022-0981(87)90168-7

[ece38753-bib-0048] Goessling, J. W. , Su, Y. , Cartaxana, P. , Maibohm, C. , Rickelt, L. F. , Trampe, E. C. L. , Walby, S. L. , Wangpraseurt, D. , Wu, X. , Ellegaard, M. , & Kuhl, M. (2018). Structure‐based optics of centric diatom frustules: modulation of the in vivo light field for efficient diatom photosynthesis. New Phytologist, 219, 122–134. 10.1111/nph.15149 29672846

[ece38753-bib-0049] Goessling, J. W. , Su, Y. , Maibohm, C. , Ellegaard, M. , & Kuhl, M. (2019). Differences in the optical properties of valve and girdle band in a centric diatom. Interface Focus, 9, 20180031. 10.1098/rsfs.2018.0031 30603064 PMC6304007

[ece38753-bib-0050] Granata, T. , Habermacher, P. , Härri, V. , & Egli, M. (2019). The influence of bio‐optical properties of Emiliania huxleyi and Tetraselmis sp. on biomass and lipid production when exposed to different light spectra and intensities of an adjustable LED array and standard light sources. SN Applied Sciences, 1. 10.1007/s42452-019-0529-x

[ece38753-bib-0051] Grobbelaar, J. U. , Nedbal, L. , & Tichy, V. (1996). Influence of high frequency light/dark fluctuations on photosynthetic characteristics of microalgae photoacclimated to different light intensities and implications for mass algal cultivation. Journal of Applied Phycology, 8, 335–343. 10.1007/BF02178576

[ece38753-bib-0052] Gronchi, E. , Johnk, K. D. , Straile, D. , Diehl, S. , & Peeters, F. (2021). Local and continental‐scale controls of the onset of spring phytoplankton blooms: Conclusions from a proxy‐based model. Global Change Biology, 27, 1976–1990. 10.1111/gcb.15521 33459454

[ece38753-bib-0053] Grosse, G. , Romanovsky, V. , Jorgenson, T. , Anthony, K. W. , Brown, J. , & Overduin, P. P. (2011). Vulnerability and feedbacks of permafrost to climate change. EOS, Transactions American Geophysical Union, 92, 73–74. 10.1029/2011eo090001

[ece38753-bib-0054] Grossman, A. R. , Schaefer, M. R. , Chiang, G. G. , & Collier, J. L. (1993). Environmental effects on the light‐harvesting complex of cyanobacteria. Journal of Bacteriology, 175, 575–582. 10.1128/jb.175.3.575-582.1993 8423132 PMC196191

[ece38753-bib-0055] Guislain, A. , Beisner, B. E. , & Köhler, J. (2018). Variation in species light acquisition traits under fluctuating light regimes: Implications for non‐equilibrium coexistence. Oikos, 128, 716–728. 10.1111/oik.05297

[ece38753-bib-0056] Gutu, A. , & Kehoe, D. M. (2012). Emerging perspectives on the mechanisms, regulation, and distribution of light color acclimation in cyanobacteria. Molecular Plant, 5, 1–13. 10.1093/mp/ssr054 21772031

[ece38753-bib-0057] Häder, D. P. , & Gao, K. (2015). Interactions of anthropogenic stress factors on marine phytoplankton. Frontiers in Environmental Science, 3, 1–14. 10.3389/fenvs.2015.00014

[ece38753-bib-0058] Häder, D. P. , Helbling, E. W. , Williamson, C. E. , & Worrest, R. C. (2011). Effects of UV radiation on aquatic ecosystems and interactions with climate change. Photochemical & Photobiological Sciences, 10, 242–260. 10.1039/c0pp90036b 21253662

[ece38753-bib-0059] Häder, D. P. , Villafane, V. E. , & Helbling, E. W. (2014). Productivity of aquatic primary producers under global climate change. Photochemical & Photobiological Sciences, 13, 1370–1392. 10.1039/c3pp50418b 25191675

[ece38753-bib-0060] Hansen, A. M. , Kraus, T. E. C. , Pellerin, B. A. , Fleck, J. A. , Downing, B. D. , & Bergamaschi, B. A. (2016). Optical properties of dissolved organic matter (DOM): Effects of biological and photolytic degradation. Limnology and Oceanography, 61, 1015–1032. 10.1002/lno.10270

[ece38753-bib-0061] Harris, G. N. , Scanlan, D. J. , & Geider, R. J. (2009). Responses of *Emiliania huxleyi* (Prymnesiophyceae) to step changes in photon flux density. European Journal of Phycology, 44, 31–48. 10.1080/09670260802233460

[ece38753-bib-0062] Harrison, J. W. , & Smith, R. E. H. (2009). Effects of ultraviolet radiation on the productivity and composition of freshwater phytoplankton communities. Photochemical & Photobiological Sciences, 8, 1218–1232. 10.1039/b902604e 19707611

[ece38753-bib-0063] Hays, G. C. , Richardson, A. J. , & Robinson, C. (2005). Climate change and marine plankton. Trends in Ecology & Evolution, 20, 337–344. 10.1016/j.tree.2005.03.004 16701390

[ece38753-bib-0064] Helbling, E. W. , Carrillo, P. , Medina‐Sanchez, J. M. , Duran, C. , Herrera, G. , Villar‐Argaiz, M. , & Villafane, V. E. (2013). Interactive effects of vertical mixing, nutrients and ultraviolet radiation: in situ photosynthetic responses of phytoplankton from high mountain lakes in Southern Europe. Biogeosciences, 10, 1037–1050. 10.5194/bg-10-1037-2013

[ece38753-bib-0065] Helbling, W. E. , Banaszak, A. T. , & Villafane, V. E. (2015). Global change feed‐back inhibits cyanobacterial photosynthesis. Scientific Reports, 5, 14514. 10.1038/srep14514 26415603 PMC4586519

[ece38753-bib-0066] Hickman, A. E. , Holligan, P. M. , Moore, C. M. , Sharples, J. , Krivtsov, V. , & Palmer, M. R. (2009). Distribution and chromatic adaptation of phytoplankton within a shelf sea thermocline. Limnology and Oceanography, 54, 525–536. 10.4319/lo.2009.54.2.0525

[ece38753-bib-0067] Hintz, N. H. , Zeising, M. , & Striebel, M. (2021). Changes in spectral quality of underwater light alter phytoplankton community composition. Limnology and Oceanography, 66(9), 3327–3337. 10.1002/lno.11882

[ece38753-bib-0068] Hoegh‐Guldberg, O. (1999). Climate change, coral bleaching and the future of the world's coral reefs. Marine and Freshwater Research, 50, 839–866. 10.1071/Mf99078

[ece38753-bib-0069] Holtrop, T. , Huisman, J. , Stomp, M. , Biersteker, L. , Aerts, J. , Grebert, T. , Partensky, F. , Garczarek, L. , & Woerd, H. J. V. (2021). Vibrational modes of water predict spectral niches for photosynthesis in lakes and oceans. Nature Ecology & Evolution, 5, 55–66. 10.1038/s41559-020-01330-x 33168993

[ece38753-bib-0070] Houser, J. N. (2006). Water color affects the stratification, surface temperature, heat content, and mean epilimnetic irradiance of small lakes. Canadian Journal of Fisheries and Aquatic Sciences, 63, 2447–2455. 10.1139/f06-131

[ece38753-bib-0071] Huisman, J. , Jonker, R. R. , Zonneveld, C. , & Weissing, F. J. (1999). Competition for light between phytoplankton species: Experimental tests of mechanistic theory. Ecology, 80, 211–222. 10.2307/176991

[ece38753-bib-0072] Huisman, J. , Sharples, J. , Stroom, J. M. , Visser, P. M. , Kardinaal, W. E. A. , Verspagen, J. M. H. , & Sommeijer, B. (2004). Changes in turbulent mixing shift competition for light between phytoplankton species. Ecology, 85, 2960–2970. 10.1890/03-0763

[ece38753-bib-0073] Huisman, J. , & Weissing, F. J. (1994). Light‐limited growth and competition for light in well‐mixed aquatic environments – An elementary model. Ecology, 75, 507–520. 10.2307/1939554

[ece38753-bib-0074] Isbell, F. , Calcagno, V. , Hector, A. , Connolly, J. , Harpole, W. S. , Reich, P. B. , Scherer‐Lorenzen, M. , Schmid, B. , Tilman, D. , van Ruijven, J. , Weigelt, A. , Wilsey, B. J. , Zavaleta, E. S. , & Loreau, M. (2011). High plant diversity is needed to maintain ecosystem services. Nature, 477, 199–202. 10.1038/nature10282 21832994

[ece38753-bib-0075] Jäger, C. G. , Diehl, S. , & Schmidt, G. M. (2008). Influence of water‐column depth and mixing on phytoplankton biomass, community composition, and nutrients. Limnology and Oceanography, 53, 2361–2373. 10.4319/lo.2008.53.6.2361

[ece38753-bib-0076] Janssen, M. , Bathke, L. , Marquardt, J. , Krumbein, W. E. , & Rhiel, E. (2001). Changes in the photosynthetic apparatus of diatoms in response to low and high light intensities. International Microbiology, 4, 27–33. 10.1007/s101230100005 11770817

[ece38753-bib-0077] Jeffrey, S. W. , & Wright, S. W. (2006). Photosynthetic pigments in marine microalgae: insights from cultures and the sea. In D. V. Subba Rao (Ed.), Algal cultures, analogues of blooms and applications (pp. 33–90). Science Publishers.

[ece38753-bib-0078] Jeon, Y. C. , Cho, C. W. , & Yun, Y. S. (2005). Measurement of microalgal photosynthetic activity depending on light intensity and quality. Biochemical Engineering Journal, 27, 127–131. 10.1016/j.bej.2005.08.017

[ece38753-bib-0079] Jin, P. , Overmans, S. , Duarte, C. M. , Agustí, S. , & Bates, A. (2019). Increasing temperature within thermal limits compensates negative ultraviolet‐B radiation effects in terrestrial and aquatic organisms. Global Ecology and Biogeography, 28, 1695–1711. 10.1111/geb.12973

[ece38753-bib-0080] Juhl, A. R. , & Krembs, C. (2010). Effects of snow removal and algal photoacclimation on growth and export of ice algae. Polar Biology, 33, 1057–1065. 10.1007/s00300-010-0784-1

[ece38753-bib-0081] Kauko, H. M. , Taskjelle, T. , Assmy, P. , Pavlov, A. K. , Mundy, C. J. , Duarte, P. , Fernandez‐Mendez, M. , Olsen, L. M. , Hudson, S. R. , Johnsen, G. , Elliott, A. , Wang, F. Y. , & Granskog, M. A. (2017). Windows in Arctic sea ice: Light transmission and ice algae in a refrozen lead. Journal of Geophysical Research‐Biogeosciences, 122, 1486–1505. 10.1002/2016jg003626

[ece38753-bib-0082] Kirk, J. T. O. (2010). Light and photosynthesis in aquatic ecosystems (3rd ed.). Cambridge University Press. 10.1017/CBO9781139168212

[ece38753-bib-0083] Köhler, J. , Wang, L. , Guislain, A. , & Shatwell, T. (2018). Influence of vertical mixing on light‐dependency of phytoplankton growth. Limnology and Oceanography, 63, 1156–1167. 10.1002/lno.10761

[ece38753-bib-0084] Koussoroplis, A. M. , Pincebourde, S. , & Wacker, A. (2017). Understanding and predicting physiological performance of organisms in fluctuating and multifactorial environments. Ecological Monographs, 87, 178–197. 10.1002/ecm.1247

[ece38753-bib-0085] Lalli, C. M. , & Parson, T. R. (1997). Biological Oceanography: An introduction (2nd ed.). Butterworth‐Heinemann. 10.1016/B978-0-7506-3384-0.X5056-7

[ece38753-bib-0086] Lannuzel, D. , Tedesco, L. , van Leeuwe, M. , Campbell, K. , Flores, H. , Delille, B. , Miller, L. , Stefels, J. , Assmy, P. , Bowman, J. , Brown, K. , Castellani, G. , Chierici, M. , Crabeck, O. , Damm, E. , Else, B. , Fransson, A. , Fripiat, F. , Geilfus, N. X. , … Wongpan, P. (2020). The future of Arctic sea‐ice biogeochemistry and ice‐associated ecosystems. Nature Climate Change, 10, 983–992. 10.1038/s41558-020-00940-4

[ece38753-bib-0087] Lavaud, J. , Strzepek, R. F. , & Kroth, P. G. (2007). Photoprotection capacity differs among diatoms: Possible consequences on the spatial distribution of diatoms related to fluctuations in the underwater light climate. Limnology and Oceanography, 52, 1188–1194. 10.4319/lo.2007.52.3.1188

[ece38753-bib-0088] Leech, D. M. , Pollard, A. I. , Labou, S. G. , & Hampton, S. E. (2018). Fewer blue lakes and more murky lakes across the continental U.S.: Implications for planktonic food webs. Limnology & Oceanography, 63, 2661–2680. 10.1002/lno.10967 31942083 PMC6961962

[ece38753-bib-0089] Lehman, J. T. (2002). Mixing patterns and plankton biomass of the St. Lawrence Great Lakes under climate change scenarios. Journal of Great Lakes Research, 28, 583–596. 10.1016/s0380-1330(02)70607-2

[ece38753-bib-0090] Lenard, T. , & Wojciechowska, W. (2013). Phytoplankton diversity and biomass during winter with and without ice cover in the context of climate change. Polish Journal of Ecology, 61, 739–748.

[ece38753-bib-0091] Lepetit, B. , Goss, R. , Jakob, T. , & Wilhelm, C. (2012). Molecular dynamics of the diatom thylakoid membrane under different light conditions. Photosynthesis Research, 111, 245–257. 10.1007/s11120-011-9633-5 21327535

[ece38753-bib-0092] Litchman, E. (2007). Resource competition and the ecological success of phytoplankton. In P. G. Falkowski , & A. H. Knoll (Eds.), Evolution of primary producers in the Sea. 10.1016/B978-012370518-1/50017-5

[ece38753-bib-0093] Litchman, E. (2008). Growth rates of phytoplankton under fluctuating light. Freshwater Biology, 44, 223–235. 10.1046/j.1365-2427.2000.00559.x

[ece38753-bib-0094] Litchman, E. , & Klausmeier, C. A. (2001). Competition of phytoplankton under fluctuating light. American Naturalist, 157, 170–187. 10.1086/318628 18707270

[ece38753-bib-0095] Luimstra, V. M. , Verspagen, J. M. H. , Xu, T. S. , Schuurmans, J. M. , & Huisman, J. (2019). Changes in water color shift competition between phytoplankton species with contrasting light‐harvesting strategies. Ecology, 101, e02951. 10.1002/ecy.2951 PMC7079016 31840230

[ece38753-bib-0096] Lyu, L. L. , Liu, G. , Shang, Y. X. , Wen, Z. D. , Hou, J. B. , & Song, K. S. (2021). Characterization of dissolved organic matter (DOM) in an urbanized watershed using spectroscopic analysis. Chemosphere, 277, 130210. 10.1016/j.chemosphere.2021.130210 33774257

[ece38753-bib-0097] Magnuson, J. J. , Robertson, D. M. , Benson, B. J. , Wynne, R. H. , Livingstone, D. M. , Arai, T. , Assel, R. A. , Barry, R. G. , Card, V. V. , Kuusisto, E. , Granin, N. G. , Prowse, T. D. , Stewart, K. M. , & Vuglinski, V. S. (2000). Historical trends in lake and river ice cover in the northern hemisphere. Science, 289, 1743–1746. 10.1126/science.289.5485.1743 10976066

[ece38753-bib-0098] Mallin, M. A. , & Paerl, H. W. (1992). Effects of variable irradiance on phytoplankton productivity in shallow estuaries. Limnology and Oceanography, 37, 54–62. 10.4319/lo.1992.37.1.0054

[ece38753-bib-0099] Markager, S. , Stedmon, C. , & Conan, P. (2004). Effects of DOM in marine ecosystems. In M. Søndergaard , & D. Thomas (Eds.), Dissolved organic matter (DOM) in aquatic ecosystems: A study of European catchments and coastal waters, The DOMAINE project.

[ece38753-bib-0100] Marra, J. (1978). Phytoplankton photosynthetic response to vertical movement in a mixed layer. Marine Biology, 46, 203–208. 10.1007/bf00390681

[ece38753-bib-0101] Marra, J. , Trees, C. C. , Bidigare, R. R. , & Barber, R. T. (2000). Pigment absorption and quantum yields in the Arabian Sea. Deep Sea Research Part II: Topical Studies in Oceanography, 47, 1279–1299. 10.1016/s0967-0645(99)00144-7

[ece38753-bib-0102] Martin, W. F. , Bryant, D. A. , & Beatty, J. T. (2018). A physiological perspective on the origin and evolution of photosynthesis. FEMS Microbiology Reviews, 42, 205–231. 10.1093/femsre/fux056 29177446 PMC5972617

[ece38753-bib-0103] Marzetz, V. , Spijkerman, E. , Striebel, M. , & Wacker, A. (2020). Phytoplankton community responses to interactions between light intensity, light variations, and phosphorus supply. Frontiers in Environmental Science, 8, 539733. 10.3389/fenvs.2020.539733

[ece38753-bib-0104] Mellard, J. P. , Yoshiyama, K. , Klausmeier, C. A. , & Litchman, E. (2012). Experimental test of phytoplankton competition for nutrients and light in poorly mixed water columns. Ecological Monographs, 82, 239–256. 10.1890/11-0273.1

[ece38753-bib-0105] Miranda, M. L. , Mustaffa, N. I. H. , Robinson, T. B. , Stolle, C. , Ribas‐Ribas, M. , Wurl, O. , & Zielinski, O. (2018). Influence of solar radiation on biogeochemical parameters and fluorescent dissolved organic matter (FDOM) in the sea surface microlayer of the southern coastal North Sea. Elementa‐Science of the Anthropocene, 6(1), 15. 10.1525/elementa.278

[ece38753-bib-0106] Montesinos, E. , Guerrero, R. , Abella, C. , & Esteve, I. (1983). Ecology and physiology of the competition for light between chlorobium‐limicola and chlorobium‐Phaeobacteroides in natural habitats. Applied and Environmental Microbiology, 46, 1007–1016. 10.1128/Aem.46.5.1007-1016.1983 16346409 PMC239512

[ece38753-bib-0107] Mouget, J. L. , Rosa, P. , & Tremblin, G. (2004). Acclimation of Haslea ostrearia to light of different spectral qualities ‐ confirmation of ‘chromatic adaptation’ in diatoms. Journal of Photochemistry and Photobiology B: Biology, 75, 1–11. 10.1016/j.jphotobiol.2004.04.002 15246344

[ece38753-bib-0108] Nann, S. , & Riordan, C. (1991). Solar spectral irradiance under clear and cloudy skies: Measurements and a semiempirical model. Journal of Applied Meteorology, 30, 447–462. https://doi.org/10.1175/1520‐0450(1991)030<0447:Ssiuca>2.0.Co;2

[ece38753-bib-0109] Neale, P. J. , Davis, R. F. , & Cullen, J. J. (1998). Interactive effects of ozone depletion and vertical mixing on photosynthesis of Antarctic phytoplankton. Nature, 392, 585–589. 10.1038/33374

[ece38753-bib-0110] Nicklisch, A. (1998). Growth and light absorption of some planktonic cyanobacteria, diatoms and Chlorophyceae under simulated natural light fluctuations. Journal of Plankton Research, 20, 105–119. 10.1093/plankt/20.1.105

[ece38753-bib-0111] Northington, R. M. , Saros, J. E. , Burpee, B. T. , & McCue, J. (2019). Changes in mixing depth reduce phytoplankton biomass in an Arctic lake: Results from a whole‐lake experiment. Arctic Antarctic and Alpine Research, 51, 533–548. 10.1080/15230430.2019.1692412

[ece38753-bib-0112] O'Dea, R. E. , Lagisz, M. , Jennions, M. D. , Koricheva, J. , Noble, D. W. A. , Parker, T. H. , Gurevitch, J. , Page, M. J. , Stewart, G. , Moher, D. , & Nakagawa, S. (2021). Preferred reporting items for systematic reviews and meta‐analyses in ecology and evolutionary biology: a PRISMA extension. Biological Reviews of the Cambridge Philosophical Society, 96, 1695–1722. 10.1111/brv.12721 33960637 PMC8518748

[ece38753-bib-0113] Parry, M. L. , Canziani, O. F. , Palutikof, J. P. , van der Linden, P. J. , & Hanson, C. E. (2007). Contribution of Working Group II to the Fourth Assessment Report of the Intergovernmental Panel on Climate Change. In Climate change 2007 – Impacts, adaptation and vulnerability. Cambridge University Press. https://www.ipcc.ch/site/assets/uploads/2018/03/ar4_wg2_full_report.pdf

[ece38753-bib-0114] Patara, L. , Vichi, M. , & Masina, S. (2012). Impacts of natural and anthropogenic climate variations on North Pacific plankton in an Earth System Model. Ecological Modelling, 244, 132–147. 10.1016/j.ecolmodel.2012.06.012

[ece38753-bib-0115] Peng, S. , Liao, H. , Zhou, T. , & Peng, S. (2017). Effects of UVB radiation on freshwater biota: A meta‐analysis. Global Ecology and Biogeography, 26, 500–510. 10.1111/geb.12552

[ece38753-bib-0116] Planas, D. , & Paquet, S. (2016). Importance of climate change‐physical forcing on the increase of cyanobacterial blooms in a small, stratified lake. Journal of Limnology, 75, 201–214. 10.4081/jlimnol.2016.1371

[ece38753-bib-0117] Popkova, A. , Mazina, S. , & Lashenova, T. Y. (2019). Phototrophic communities of Ahshtyrskaya Cave in the condition of artificial light. Ecologica Montenegrina, 23, 8–19. 10.37828/em.2019.23.2

[ece38753-bib-0118] Ragni, M. (2004). Light as an information carrier underwater. Journal of Plankton Research, 26, 433–443. 10.1093/plankt/fbh044

[ece38753-bib-0119] Raven, J. A. , & Cockell, C. S. (2006). Influence on photosynthesis of starlight, moonlight, planetlight, and light pollution (reflections on photosynthetically active radiation in the universe). Astrobiology, 6, 668–675. 10.1089/ast.2006.6.668 16916290

[ece38753-bib-0120] Raven, J. A. , & Geider, R. J. (2003). Adaptation, acclimation and regulation in algal photosynthesis. In A. W. D. Larkum , S. E. Douglas , & J. A. Raven (Eds.), Photosynthesis in algae. 10.1007/978-94-007-1038-2_17

[ece38753-bib-0121] Rensing, S. A. , Sheerin, D. J. , & Hiltbrunner, A. (2016). Phytochromes: More than meets the eye. Trends in Plant Science, 21, 543–546. 10.1016/j.tplants.2016.05.009 27270335

[ece38753-bib-0122] Retkute, R. , Smith‐Unna, S. E. , Smith, R. W. , Burgess, A. J. , Jensen, O. E. , Johnson, G. N. , Preston, S. P. , & Murchie, E. H. (2015). Exploiting heterogeneous environments: does photosynthetic acclimation optimize carbon gain in fluctuating light? Journal of Experimental Botany, 66, 2437–2447. 10.1093/jxb/erv055 25788730 PMC4629418

[ece38753-bib-0123] Richardson, K. , Beardall, J. , & Raven, J. A. (1983). Adaptation of unicellular algae to irradiance – An analysis of strategies. New Phytologist, 93, 157–191. 10.1111/j.1469-8137.1983.tb03422.x

[ece38753-bib-0124] Rocap, G. , Larimer, F. W. , Lamerdin, J. , Malfatti, S. , Chain, P. , Ahlgren, N. A. , Arellano, A. , Coleman, M. , Hauser, L. , Hess, W. R. , Johnson, Z. I. , Land, M. , Lindell, D. , Post, A. F. , Regala, W. , Shah, M. , Shaw, S. L. , Steglich, C. , Sullivan, M. B. , … Chisholm, S. W. (2003). Genome divergence in two Prochlorococcus ecotypes reflects oceanic niche differentiation. Nature, 424, 1042–1047. 10.1038/nature01947 12917642

[ece38753-bib-0125] Rockwell, N. C. , Duanmu, D. , Martin, S. S. , Bachy, C. , Price, D. C. , Bhattacharya, D. , Worden, A. Z. , & Lagarias, J. C. (2014). Eukaryotic algal phytochromes span the visible spectrum. Proceedings of the National Academy of Sciences of the United States of America, 111, 3871–3876. 10.1073/pnas.1401871111 24567382 PMC3956157

[ece38753-bib-0126] Roulet, N. , & Moore, T. R. (2006). Environmental chemistry: Browning the waters. Nature, 444, 283–284. 10.1038/444283a 17108948

[ece38753-bib-0127] Ryther, J. H. (1956). Photosynthesis in the Ocean as a function of light intensity. Limnology and Oceanography, 1, 61–70. 10.4319/lo.1956.1.1.0061

[ece38753-bib-0128] Sánchez‐Saavedra, M. P. , & Voltolina, D. (2002). Effect of photon fluence rates of white and blue‐green light on growth efficiency and pigment content of three diatom species in batch cultures. Ciencias Marinas, 28, 273–279. 10.7773/cm.v28i3.225

[ece38753-bib-0129] Saros, J. E. , Stone, J. R. , Pederson, G. T. , Slemmons, K. E. H. , Spanbauer, T. , Schliep, A. , Cahl, D. , Williamson, C. E. , & Engstrom, D. R. (2012). Climate‐induced changes in lake ecosystem structure inferred from coupled neo‐ and paleoecological approaches. Ecology, 93, 2155–2164. 10.1890/11-2218.1 23185877

[ece38753-bib-0130] Schenck, H. (1957). On the focusing of sunlight by ocean waves. Journal of the Optical Society of America, 47, 653–657. 10.1364/josa.47.000653

[ece38753-bib-0131] Schubert, H. , Sagert, S. , & Forster, R. M. (2001). Evaluation of the different levels of variability in the underwater light field of a shallow estuary. Helgoland Marine Research, 55, 12–22. 10.1007/s101520000064

[ece38753-bib-0132] Schwaderer, A. S. , Yoshiyama, K. , Pinto, P. T. , Swenson, N. G. , Klausmeier, C. A. , & Litchman, E. (2011). Eco‐evolutionary differences in light utilization traits and distributions of freshwater phytoplankton. Limnology and Oceanography, 56, 589–598. 10.4319/lo.2011.56.2.0589

[ece38753-bib-0133] Shigesada, N. , & Okubo, A. (1981). Analysis of the self‐shading effect on algal vertical‐distribution in natural‐waters. Journal of Mathematical Biology, 12, 311–326. 10.1007/Bf00276919

[ece38753-bib-0134] Smith, R. C. (1989). Ozone, middle ultraviolet radiation and the aquatic environment. Photochemistry and Photobiology, 50, 459–468. 10.1111/j.1751-1097.1989.tb05550.x

[ece38753-bib-0135] Smyth, R. L. , Akan, C. , Tejada‐Martínez, A. , & Neale, P. J. (2017). Quantifying phytoplankton productivity and photoinhibition in the Ross Sea Polynya with large eddy simulation of Langmuir circulation. Journal of Geophysical Research: Oceans, 122, 5545–5565. 10.1002/2017JC012747

[ece38753-bib-0136] Somavilla, R. , Gonzalez‐Pola, C. , & Fernandez‐Diaz, J. (2017). The warmer the ocean surface, the shallower the mixed layer. How much of this is true? Journal of Geophysical Research: Oceans, 122, 7698–7716. 10.1002/2017JC013125 29201584 PMC5699439

[ece38753-bib-0137] Steiner, N. , Azetsu‐Scott, K. , Hamilton, J. , Hedges, K. , Hu, X. M. , Janjua, M. Y. , Lavoie, D. , Loder, J. , Melling, H. , Merzouk, A. , Perrie, W. , Peterson, I. , Scarratt, M. , Sou, T. , & Tallmann, R. (2015). Observed trends and climate projections affecting marine ecosystems in the Canadian Arctic. Environmental Reviews, 23, 191–239. 10.1139/er-2014-0066

[ece38753-bib-0138] Stomp, M. , Huisman, J. , de Jongh, F. , Veraart, A. J. , Gerla, D. , Rijkeboer, M. , Ibelings, B. W. , Wollenzien, U. I. , & Stal, L. J. (2004). Adaptive divergence in pigment composition promotes phytoplankton biodiversity. Nature, 432, 104–107. 10.1038/nature03044 15475947

[ece38753-bib-0139] Stomp, M. , Huisman, J. , Stal, L. J. , & Matthijs, H. C. (2007). Colorful niches of phototrophic microorganisms shaped by vibrations of the water molecule. ISME Journal, 1, 271–282. 10.1038/ismej.2007.59 18043638

[ece38753-bib-0140] Stomp, M. , Huisman, J. , Voros, L. , Pick, F. R. , Laamanen, M. , Haverkamp, T. , & Stal, L. J. (2007). Colourful coexistence of red and green picocyanobacteria in lakes and seas. Ecology Letters, 10, 290–298. 10.1111/j.1461-0248.2007.01026.x 17355568

[ece38753-bib-0141] Straka, L. , & Rittmann, B. E. (2018). Light‐dependent kinetic model for microalgae experiencing photoacclimation, photodamage, and photodamage repair. Algal Research‐Biomass Biofuels and Bioproducts, 31, 232–238. 10.1016/j.algal.2018.02.022

[ece38753-bib-0142] Striebel, M. , Behl, S. , Diehl, S. , & Stibor, H. (2009). Spectral niche complementarity and carbon dynamics in pelagic ecosystems. American Naturalist, 174, 141–147. 10.1086/599294 19456261

[ece38753-bib-0143] Strzepek, R. F. , & Harrison, P. J. (2004). Photosynthetic architecture differs in coastal and oceanic diatoms. Nature, 431, 689–692. 10.1038/nature02954 15470428

[ece38753-bib-0144] Sydeman, W. J. , Garcia‐Reyes, M. , Schoeman, D. S. , Rykaczewski, R. R. , Thompson, S. A. , Black, B. A. , & Bograd, S. J. (2014). Climate change. Climate change and wind intensification in coastal upwelling ecosystems. Science, 345, 77–80. 10.1126/science.1251635 24994651

[ece38753-bib-0145] Szymańska, R. , Ślesak, I. , Orzechowska, A. , & Kruk, J. (2017). Physiological and biochemical responses to high light and temperature stress in plants. Environmental and Experimental Botany, 139, 165–177. 10.1016/j.envexpbot.2017.05.002

[ece38753-bib-0146] Thrane, J. E. , Hessen, D. O. , & Andersen, T. (2014). The absorption of light in lakes: Negative impact of dissolved organic carbon on primary productivity. Ecosystems, 17, 1040–1052. 10.1007/s10021-014-9776-2

[ece38753-bib-0147] Ting, C. S. , Rocap, G. , King, J. , & Chisholm, S. W. (2002). Cyanobacterial photosynthesis in the oceans: The origins and significance of divergent light‐harvesting strategies. Trends in Microbiology, 10, 134–142. 10.1016/s0966-842x(02)02319-3 11864823

[ece38753-bib-0148] van Leeuwe, M. A. , van Sikkelerus, B. , Gieskes, W. W. C. , & Stefels, J. (2005). Taxon‐specific differences in photoacclimation to fluctuating irradiance in an Antarctic diatom and a green flagellate. Marine Ecology Progress Series, 288, 9–19. 10.3354/meps288009

[ece38753-bib-0149] Vasconcelos, F. R. , Diehl, S. , Rodriguez, P. , Hedstrom, P. , Karlsson, J. , & Bystrom, P. (2016). Asymmetrical competition between aquatic primary producers in a warmer and browner world. Ecology, 97, 2580–2592. 10.1002/ecy.1487 27859128

[ece38753-bib-0150] Vesk, M. , & Jeffrey, S. W. (1977). Effect of blue‐green light on photosynthetic pigments and chloroplast structure in unicellular marine‐algae from 6 classes. Journal of Phycology, 13, 280–288. 10.1111/j.1529-8817.1977.tb02928.x

[ece38753-bib-0151] Villafane, V. E. , Paczkowska, J. , Andersson, A. , Romero, C. D. , Valinas, M. S. , & Helbling, E. W. (2018). Dual role of DOM in a scenario of global change on photosynthesis and structure of coastal phytoplankton from the South Atlantic Ocean. Science of the Total Environment, 634, 1352–1361. 10.1016/j.scitotenv.2018.04.121 29710635

[ece38753-bib-0152] Vizzo, J. I. , Cabrerizo, M. J. , Helbling, E. W. , & Villafane, V. E. (2021). Extreme and gradual rainfall effects on winter and summer estuarine phytoplankton communities from Patagonia (Argentina). Marine Environmental Research, 163, 105235. 10.1016/j.marenvres.2020.10523 33338796

[ece38753-bib-0153] Voet, D. , & Voet, J. G. (2010). Biochemistry (4th ed.). Wiley‐VCH.

[ece38753-bib-0154] Wahl, B. , & Peeters, F. (2014). Effect of climatic changes on stratification and deep‐water renewal in Lake Constance assessed by sensitivity studies with a 3D hydrodynamic model. Limnology and Oceanography, 59, 1035–1052. 10.4319/lo.2014.59.3.1035

[ece38753-bib-0155] Walsh, P. , & Legendre, L. (1983). Photosynthesis of natural phytoplankton under high‐frequency light fluctuations simulating those induced by sea‐surface waves. Limnology and Oceanography, 28, 688–697. 10.4319/lo.1983.28.4.0688

[ece38753-bib-0156] Weissing, F. J. , & Huisman, J. (1994). Growth and competition in a light gradient. Journal of Theoretical Biology, 168, 323–336. 10.1006/jtbi.1994.1113

[ece38753-bib-0157] Weyhenmeyer, G. A. , & Karlsson, J. (2009). Nonlinear response of dissolved organic carbon concentrations in boreal lakes to increasing temperatures. Limnology and Oceanography, 54, 2513–2519. 10.4319/lo.2009.54.6_part_2.2513

[ece38753-bib-0158] Weyhenmeyer, G. A. , Müller, R. A. , Norman, M. , & Tranvik, L. J. (2015). Sensitivity of freshwaters to browning in response to future climate change. Climatic Change, 134, 225–239. 10.1007/s10584-015-1514-z

[ece38753-bib-0159] Williamson, C. J. , Cook, J. , Tedstone, A. , Yallop, M. , McCutcheon, J. , Poniecka, E. , Campbell, D. , Irvine‐Fynn, T. , McQuaid, J. , Tranter, M. , Perkins, R. , & Anesio, A. (2020). Algal photophysiology drives darkening and melt of the Greenland Ice Sheet. Proceedings of the National Academy of Sciences of the United States of America, 117, 5694–5705. 10.1073/pnas.1918412117 32094168 PMC7084142

[ece38753-bib-0160] Williamson, C. E. , Neale, P. J. , Hylander, S. , Rose, K. C. , Figueroa, F. L. , Robinson, S. A. , Hader, D. P. , Wangberg, S. A. , & Worrest, R. C. (2019). The interactive effects of stratospheric ozone depletion, UV radiation, and climate change on aquatic ecosystems. Photochemical & Photobiological Sciences, 18, 717–746. 10.1039/c8pp90062k 30810561

[ece38753-bib-0161] Williamson, C. E. , Zepp, R. G. , Lucas, R. M. , Madronich, S. , Austin, A. T. , Ballaré, C. L. , Norval, M. , Sulzberger, B. , Bais, A. F. , McKenzie, R. L. , Robinson, S. A. , Häder, D.‐P. , Paul, N. D. , & Bornman, J. F. (2014). Solar ultraviolet radiation in a changing climate. Nature Climate Change, 4, 434–441. 10.1038/nclimate2225

[ece38753-bib-0162] Wu, Y. P. , Yue, F. R. , Xu, J. T. , & Beardall, J. (2017). Differential photosynthetic responses of marine planktonic and benthic diatoms to ultraviolet radiation under various temperature regimes. Biogeosciences, 14, 5029–5037. 10.5194/bg-14-5029-2017

