# Supplementary material for: Ecological impacts of photosynthetic light harvesting in changing aquatic environments: A systematic literature map
Source: Ecol Evol. 2022 Mar 22;12(3):e8753. doi: 10.1002/ece3.8753 (PMC8939368; doi:10.1002/ece3.8753)
Supplement: Supplementary file 1 — Appendix S1 [file ECE3-12-e8753-s002.docx]

Ecological impacts of photosynthetic light harvesting in changing aquatic environments: A systematic literature map

Nils Hendrik Hintz^1*^, Brian Schulze^2^, Alexander Wacker^2^, Maren Striebel^1^

^1^Institute for Chemistry and Biology of the Marine Environment (ICBM), Carl von Ossietzky University of Oldenburg, Wilhelmshaven, Germany

^2^ Zoological Institute and Museum, University of Greifswald, Greifswald, Germany

*Correspondence: nihehintz@gmail.com

# Supplementary Material 1: Search terms

In accordance with Item 5 of the PRISMA-EcoEvo Statement (O’Dea et al. 2021), here we report the type of search and search terms as well as the used database. We conducted comprehensive electronic searches for published resources in Web of Science on 15^th^ of June 2021 covering all published data for each of the three main objectives for all published work, and all years (since 1945). Suitable articles were identified by “topic” i.e., keywords in titles, abstracts, and author keywords of those records. The resulting records were imported to EndNote version X8 (Clarivate).

The full reference list of all included studies can be accessed online at Dryad (https://doi.org/10.5061/dryad.7h44j0zw5).

**Reference**

O'Dea, R. E., M. Lagisz, M. D. Jennions, J. Koricheva, D. W. A. Noble, T. H. Parker, J. Gurevitch, M. J. Page, G. Stewart, D. Moher, and S. Nakagawa. 2021. Preferred reporting items for systematic reviews and meta-analyses in ecology and evolutionary biology: a PRISMA extension. Biol Rev Camb Philos Soc.

# Search terms

The following search terms and Boolean characters were used to identify articles related to the main objectives. The search was conducted separately per objective.

## Objective 1

TS= ((phytoplankton OR alga* OR microalga*) AND (light OR spectrum OR spectral OR irradiance OR intensity OR wavelength$ OR “light color”) AND (photosynthe* OR absorb* OR resource$ OR acclimat* OR photoacclimat* OR adaptation OR response$ OR select*) NOT (macroalga* OR coral* OR seaweed OR toxi* OR terrestrial)) AND SU=( Biodiversity & Conservation OR Biophysics OR Environmental Sciences & Ecology OR Evolutionary Biology OR Marine & Freshwater Biology OR Biotechnology & Applied Microbiology) AND (TI=(light) OR AK=(light))

## Objective 2

TS= ((phytoplankton OR alga* OR microalga*) AND (photosynthe* OR light OR spectrum OR spectral OR irradiance OR intensity OR wavelength$ OR browning OR color) AND (fluctuat* OR repetitive OR repeating OR repetition ) AND (ecology* OR competition OR competing OR complementary OR “niche differentiation” OR *diversity OR coexistence OR coexisting OR “luxury consumption”) NOT (macroalga* OR coral* OR seaweed OR toxi* OR terrestrial)) AND SU=( Biodiversity & Conservation OR Biophysics OR Environmental Sciences & Ecology OR Evolutionary Biology OR Marine & Freshwater Biology OR Biotechnology & Applied Microbiology)

## Objective 3

TS=((phytoplankton OR alga* OR microalga*) AND (photosynthe* OR light OR spectrum OR spectral OR irradiance OR intensity OR wavelength$ OR browning OR color) AND (climate OR “climatic change$” OR “climate change$” OR “global warming” OR “global change$”) AND (stratification OR wind OR “mixed layer” OR “water column” OR “dissolved organic carbon” OR doc OR dom OR cDOM OR “darkening” OR “sediment resuspension” OR eutrophicate* OR uv OR “ultraviolet” OR “uvb” OR “uva” OR ozone OR ice OR “sea ice” OR “ice cover” OR melting OR “ice transmittance” Or “ice transmission” OR “artificial light” OR “artificial light at night” OR “light pollution” OR “polluting light” OR streetlight OR ALAN) NOT (macroalga* OR coral* OR seaweed OR toxi* OR terrestrial)) AND SU=( Biodiversity & Conservation OR Biophysics OR Environmental Sciences & Ecology OR Evolutionary Biology OR Marine & Freshwater Biology OR Biotechnology & Applied Microbiology)
