# Supplementary material for: Ecological impacts of photosynthetic light harvesting in changing aquatic environments: A systematic literature map
Source: Ecol Evol. 2022 Mar 22;12(3):e8753. doi: 10.1002/ece3.8753 (PMC8939368; doi:10.1002/ece3.8753)
Supplement: Supplementary file 2 — Appendix S2 [file ECE3-12-e8753-s003.docx]

Ecological impacts of photosynthetic light harvesting in changing aquatic environments: A systematic literature map

Nils Hendrik Hintz^1*^, Brian Schulze^2^, Alexander Wacker^2^, Maren Striebel^1^

^1^Institute for Chemistry and Biology of the Marine Environment (ICBM), Carl von Ossietzky University of Oldenburg, Wilhelmshaven, Germany

^2^ Zoological Institute and Museum, University of Greifswald, Greifswald, Germany

*Correspondence: nihehintz@gmail.com

# Supplementary Material 2: Flow Chart

In accordance with Item 19 of the PRISMA-EcoEvo Statement (O’Dea et al. 2021), here we report the number of studies screened and the number of studies excluded at each stage of screening. This is presented in a classic PRISMA-style flowchart format following Page et al. (2020). For each of the three objectives a separate search and screening process was conducted. Reasons for exclusion of identified records (Step: “Record screened”) were unsuitability of the study. This was e.g., if a study did not target the respective objective. No automation tools were used for exclusion of studies. Additional useful and criteria fitting studies which were known to the authors, listed in other studies reference lists, or identified within the respective other objectives, were added manually in the last step. Finally, the number of studies per objective is presented which were referred to within the main manuscript.

The initial search resulted in a total of 3357 records (Objective 1 (O1): 2138; Objective 2 (O2): 241; Objective 3 (O3): 978) i.e., a total of 3128 individual articles due to duplication across the objectives. Title screening led to 1495 records (O1: 544; O2: 77; O3: 874) i.e., 1421 individual articles. Abstract screening resulted in 546 records (O1: 303; O2: 38; O3: 205), i.e., 520 individual articles. With inclusion of additional articles, a total of 675 records (O1: 361; O2: 59; O3: 255) i.e., 640 individual articles were retrieved and considered for mapping.

The full reference list of all included studies can be accessed online at Dryad (https://doi.org/10.5061/dryad.7h44j0zw5).

**References**

O'Dea, R. E., M. Lagisz, M. D. Jennions, J. Koricheva, D. W. A. Noble, T. H. Parker, J. Gurevitch, M. J. Page, G. Stewart, D. Moher, and S. Nakagawa. 2021. Preferred reporting items for systematic reviews and meta-analyses in ecology and evolutionary biology: a PRISMA extension. Biol Rev Camb Philos Soc.

Page, M.J., McKenzie, J.E, Bossuyt, P.M., Boutron, I., Hoffmann, T.C., Mulrow, C.D., et al. The PRISMA 2020 statement: an updated guideline for reporting systematic reviews. BMJ 2021;372:n71. doi: 10.1136/bmj.n71

**Objective 1 Identification of studies via databases and registers**

Records removed *before screening*:

Duplicate records removed (n = 0)

Records marked as ineligible by automation tools (n = )

Records removed for other reasons (n = 0)

Records identified from*:

Databases (n = 1)

(n = 2138)

**Identification**

Records excluded**

manual title screening (n =1594)
manual abstract screening (n=241)

Records screened

(n = 2138)

**Screening**

Reports sought for retrieval

(n = 303)

Reports not retrievable

(n = 0)

Reports manually added

(n= 59)

Studies assessed for review developing

(n = 362)

**Included**

**Objective 2 Identification of studies via databases and registers**

Records removed *before screening*:

Duplicate records removed (n = 0)

Records marked as ineligible by automation tools (n = )

Records removed for other reasons (n = 0)

Records identified from*:

Databases (n = 1)

(n = 241)

**Identification**

Records excluded**

manual title screening (n =164)
manual abstract screening (n = 39))

Records screened

(n = 241)

**Screening**

Reports sought for retrieval

(n = 38)

Reports not retrievable

(n = 0)

Reports manually added

(n = 20)

Studies assessed for review developing

(n = 58)

**Included**

**Objective 3 Identification of studies via databases and registers**

Records removed *before screening*:

Duplicate records removed (n = 0)

Records marked as ineligible by automation tools (n = )

Records removed for other reasons (n = 0)

Records identified from*:

Databases (n = 1)

(n = 978)

**Identification**

Records excluded**

manual title screening (n = 103)
manual abstract screening (n = 664)

Records screened

(n = 978)

**Screening**

Reports sought for retrieval

(n = 205)

Reports not retrievable

(n = 0)

Reports manually added

(n = 50)

Studies assessed for review developing

(n = 255)

**Included**

*Consider, if feasible to do so, reporting the number of records identified from each database or register searched (rather than the total number across all databases/registers).

**If automation tools were used, indicate how many records were excluded by a human and how many were excluded by automation tools.

*From:*  Page MJ, McKenzie JE, Bossuyt PM, Boutron I, Hoffmann TC, Mulrow CD, et al. The PRISMA 2020 statement: an updated guideline for reporting systematic reviews. BMJ 2021;372:n71. doi: 10.1136/bmj.n71

For more information, visit: http://www.prisma-statement.org/
